# *In silico* genomic insights into aspects of food safety and defense mechanisms of a potentially probiotic *Lactobacillus pentosus* MP-10 isolated from brines of naturally fermented Aloreña green table olives

**DOI:** 10.1371/journal.pone.0176801

**Published:** 2017-06-26

**Authors:** Hikmate Abriouel, Beatriz Pérez Montoro, María del Carmen Casado Muñoz, Charles W. Knapp, Antonio Gálvez, Nabil Benomar

**Affiliations:** 1 Área de Microbiología, Departamento de Ciencias de la Salud, Facultad de Ciencias Experimentales, Universidad de Jaén, Jaén, Spain; 2 Department of Civil and Environmental Engineering, University of Strathclyde, Glasgow, Scotland, United Kingdom; Defense Threat Reduction Agency, UNITED STATES

## Abstract

*Lactobacillus pentosus* MP-10, isolated from brines of naturally fermented Aloreña green table olives, exhibited high probiotic potential. The genome sequence of *L*. *pentosus* MP-10 is currently considered the largest genome among lactobacilli, highlighting the microorganism’s ecological flexibility and adaptability. Here, we analyzed the complete genome sequence for the presence of acquired antibiotic resistance and virulence determinants to understand their defense mechanisms and explore its putative safety in food. The annotated genome sequence revealed evidence of diverse mobile genetic elements, such as prophages, transposases and transposons involved in their adaptation to brine-associated niches. *In-silico* analysis of *L*. *pentosus* MP-10 genome sequence identified a CRISPR (clustered regularly interspaced short palindromic repeats)/cas (CRISPR-associated protein genes) as an immune system against foreign genetic elements, which consisted of six arrays (4–12 repeats) and eleven predicted *cas* genes [CRISPR1 and CRISPR2 consisted of 3 (Type II-C) and 8 (Type I) genes] with high similarity to *L*. *pentosus* KCA1. Bioinformatic analyses revealed *L*. *pentosus* MP-10 to be absent of acquired antibiotic resistance genes, and most resistance genes were related to efflux mechanisms; no virulence determinants were found in the genome. This suggests that *L*. *pentosus* MP-10 could be considered safe and with high-adaptation potential, which could facilitate its application as a starter culture and probiotic in food preparations.

## Introduction

Lactobacilli are ubiquitous in the environment and food production (reviewed in [1]), and they are also part of intestinal, vaginal and oral microbiota [2]. As members of the lactic acid bacteria (LAB), they have been used in food fermentation processes for millennia; however, in the last decade more attention has focused on their probiotic capacity. Thus, when consumed, sufficient live cultures may benefit the host’s health [3]. Lactobacilli and bifidobacteria represent the main LAB probiotics traditionally isolated from human sources (e.g., milk and intestinal tract). However, probiotic LAB from non-dairy origin, such as fruits and vegetables, have increased in the last few years due to increasing frequencies of lactose intolerance, dyslipidemia, allergy and vegetarianism among people [4–6]. Furthermore, those food matrices are characterized by intrinsic physico-chemical properties that mimic conditions in the gastrointestinal tract, since probiotic bacteria from vegetables or fruits possess mechanisms for adherence to surfaces similarly as they would on the intestinal surface, along with their tolerance to acids and several other stresses. As such, several studies have focused on the selection of new probiotic candidates [7, 8] with LAB abundances between 102–10^4^ CFU/g on fruit and vegetable surfaces [9, 10] and 106–10^8^ CFU/g in fermented foods [11, 12].

Along with the probiotic features of some lactobacilli strains, aspects of food safety should be considered as both properties are inherently linked to the specific strains and host susceptibility [13]. Although many *Lactobacillus* spp. are recognized as GRAS (Generally Regarded As Safe; in the USA) or have attained the QPS (Qualified Presumption of Safety; for the European Commission; European Food Safety Authority “EFSA”) [14] status, probiotic properties and safety aspects of the intended probiotic bacterium should be thoroughly analyzed at genomic scale. Thus, probiogenomics [15] could offer a novel approach to verify the absence of genes related to virulence or antibiotic-resistance transferability and the presence of genes involved in health-promotion.

The complete genome of a potential probiotic *Lactobacillus pentosus* MP-10, isolated from brines of naturally fermented Aloreña green table olives, was initially sequenced in 2011 [16] and completed in 2016 [17]; in this study, it was re-annotated to provide deeper insight into its defense mechanisms—e.g., antibiotic-resistance and virulence determinants. In this sense, bioinformatic tools could provide a greater sense of the microorganism’s safety in food preparations.

## Results and discussion

### General genomic features of a probiotic *Lactobacillus pentosus* MP-10

**Fig 1:**
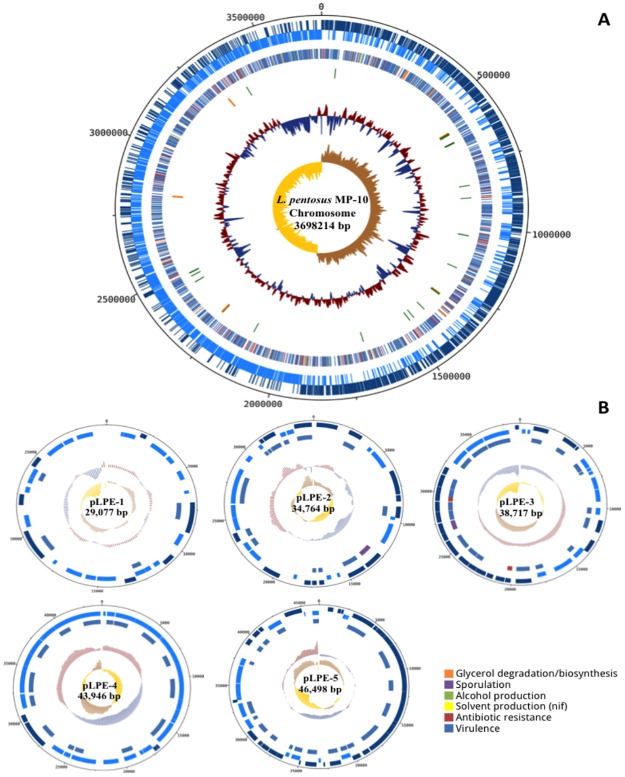
Circular representation of the *Lactobacillus pentosus* MP-10 chromosome (A) and 5 plasmids (B). (A) The circles from outside to inside are the annotated CDS elements in forward orientation, the annotated CDS elements in the reverse orientation, several COG functions, the structural RNA, the GC content and the GC screw. (B) The circles from outside to inside of each plasmid are the annotated CDS elements in forward orientation, the annotated CDS elements in the revers orientation, several COG functions, the GC content and the GC screw.

*Lactobacillus pentosus* MP-10 has the largest genome among lactobacilli considered to date, which may reflect the bacterium’s ecological flexibility and adaptability. The single circular chromosome of *L*. *pentosus* MP-10 consisted of 3,698,214 bp, with an estimated mol% G+C content of 46.32% and 5 plasmids ranging 29–46 kb [17], as represented in Fig 1. The annotated genome sequence (Fig 1A) revealed 3,558 open reading frames (ORFs), of which 84.5% (2,971) were attributed to a COG (Cluster of Orthologous Groups) family and/or were given a functional description; such number exceeded the estimate of protein-coding genes in LAB, of 1,700–2,800 genes [18], and also in *L*. *pentosus* strains—such as *L*. *pentosus* IG1 from Spanish-style fermented green olives (3,133 ORFs) [19] and *L*. *pentosus* KCA1 isolated from a vaginal source (2,992 ORFs) [20]. The genetic variability among *L*. *pentosus* strains may be based on their ecological niches as reported by O´Sullivan et al. [21], which compared genomes from different niches. Thus, lactobacilli isolated from fermented olives showed a higher number of predicted ORFs than other sources. Furthermore, ecological adaptability to fermentation is reflected by the presence of additional plasmids in *L*. *pentosus* MP-10 (five plasmids; Fig 1B) and seven plasmids in *L*. *pentosus* IG1 [19]; plasmids were absent in *L*. *pentosus* KCA1 [20]. This suggests that plasmid-borne genes mediate the persistence of lactobacilli in olive fermentation; however, this hypothesis requires further studies for confirmation.

S1 Fig (Supplemental Material) shows the cellular component, the molecular function and the biological process frequencies predicted in *L*. *pentosus* MP-10. Among the GO (Gene Ontology) terms, 230 belonged to transcription (DNA-templated), 104 transcription regulation (DNA-templated), 77 to phosphoenolpyruvate-dependent sugar phosphotransferase system, 73 to carbohydrate metabolism, 65 to response to antibiotics, 60 to cell-wall organization, 54 to transport, 48 to sporulation, 33 to glycolytic process and gluconeogenesis, and 12 to defense responses, et al. (S1 Fig).

**Fig 2:**
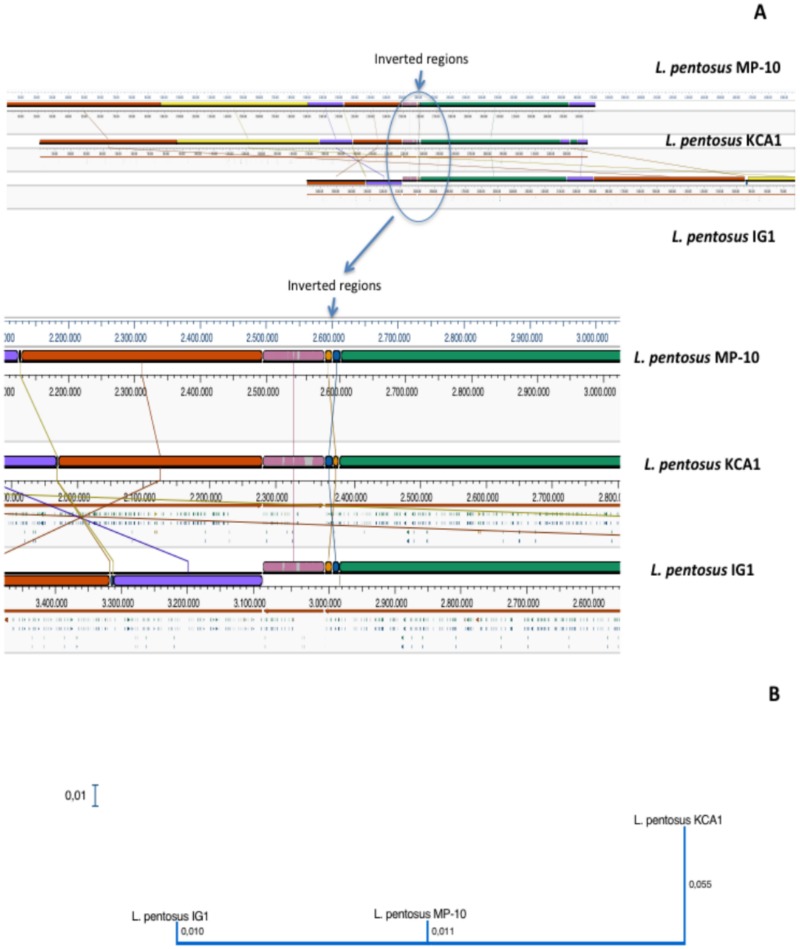
Mauve visualization of whole genome alignment of *L*. *pentosus* MP-10 with *L*. *pentosus* IG1 and *L*. *pentosus* KCA1 (A) and the phylogenetic tree (B).

Comparison of ORFs sequences among *L*. *pentosus* MP-10, *L*. *pentosus* KCA1, and *L*. *pentosus* IG1 (aligned by MAUVE algorithm) showed that the synteny of genes was similar (Fig 2A), although inversion and rearrangements among all *L*. *pentosus* strains occurred (Fig 2A). Inversion and rearrangement are the main evolutionary phenomena observed among *L*. *pentosus* strains and provide a complete picture of genetic differences among the strains colonizing different ecological niches. The phylogenetic distance between *L*. *pentosus* MP-10 and *L*. *pentosus* IG1, both isolated from olives, was lower than with *L*. *pentosus* KCA1 from vagina (Fig 2B), thus *L*. *pentosus* MP-10 was phylogenetically more closely related with *L*. *pentosus* IG1.

### Defense mechanisms of *Lactobacillus pentosus* MP-10

**Fig 3:**
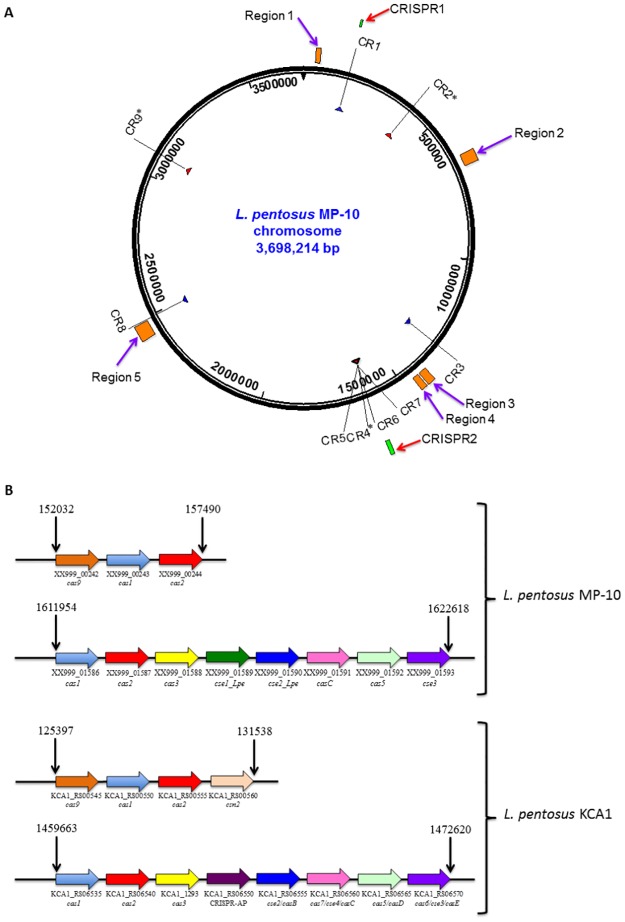
Localization of CRISPR elements and prophage regions in *L*. *pentosus* MP-10 genome. (A) Schematic view of the genomic locations of CRISPR arrays (CR) numbered according to the CRISPRdb database. The locations of associated *cas* Operons (CRISPR1 and CRISPR2) and prophage regions (Region 1, Region 2, Region 3, Region 4 and Region 5), which are numbered according to PHAST are indicated. The asteriscs indicated the questionable CRISPR arrays. (B) Organization of the *cas* operons (CRISPR1 and CRISPR2) of *L*. *pentosus* MP-10 and *L*. *pentosus* KCA1. The same color was used for homologous *cas* genes. The start and end positions are indicated in each case.

Among the defense mechanisms revealed in the *L*. *pentosus* MP-10 genome sequence by *in silico* analysis, 12 genes were found to be involved in defense responses to viruses and bacteria. Further, we identified the presence of two CRISPR systems: CRISPR1 and CRISPR2 [17] that represent an acquired and adaptive immune system providing protection against mobile genetic elements (i.e., viruses, transposable elements and conjugative plasmids) [22, 23]. In general, a CRISPR mechanism depends on a leader sequence, CRISPR array and CRISPR associated protein responsible genes (*cas* genes) in bacteria since the expression of CRISPR array could be constitutive or inducible [24, 25]. Analysis carried out with the CRISPRs finder program showed that *L*. *pentosus* MP-10 genome possessed genes that encoded nine potential CRISPR arrays (CR) between 159,766 and 3,085,353 bp distributed on the entire whole genome (Fig 3A): six were confirmed CRISPRs, and three were questionable CRISPRs (Fig 3A, Table 1). This may reflect chromosomal plasticity as a means of increasing fitness or changing ecological lifestyles.

**Table 1:** Characteristics of CRISPR arrays detected in *Lactobacillus pentosus* MP-10 and other lactobacilli genomes by using CRISPR finder program.

Strains	CRISPR array	Start position	End position	CRISPR length	Number of repeats	DR consensus**
*L*. *pentosus* MP-10	CR1	159072	159766	694	11	GTCTTGAATAGTAGTCATATCAAACAGGTTTAGAAC
	CR2*	409315	09451	136	2	CAATCCGTAGCTAAGTCACGTGCACCTGTTT
	CR3	1319339	1319917	578	10	GGATCACCCCCGCATACACGGGGAACAG
	CR4*	1609619	1609708	89	2	GGATCACCCCCGCATACGCGGGGAACAG
	CR5	1610289	1610562	273	5	GGATCACCCCCGCATACGCGGGGAACAG
	CR6	1610698	1611397	699	12	GGATCACCCCCGCATACGCGGGGAACAG
	CR7	1614018	1614531	513	9	ATCACCCCCGCATACACGGGGAACAG
	CR8	2492891	2493112	221	4	TACAGGTGCAGTGGTTGGTGCAGT
	CR9*	3085283	3085353	70	2	CTAGTTGCGGTACTTGAAGCCTT
*L*. *pentosus* KCA1	NZ_CM001538_1	131563	132851	1288	20	GTCTTGAATAGTAGTCATATCAAACAGGTTTAGAAC
	NZ_CM001538_2	1239838	1241143	1305	22	GGATCACCCCCGCATACGCGGGGAACAG
	NZ_CM001538_3	1456695	1459106	2411	40	GGATCACCCCCGCATACGCGGGGAACAG
	NZ_CM001538_4	1461724	1462549	825	14	AGGATCACCCCCGCATACACGGGGAATAG
	NZ_CM001538_5	1462701	1463218	517	9	AGGATCACCCCCGCATACACGGGGAATAG
	NZ_CM001538_6	1463351	1464538	1187	20	AGGATCACCCCCGCATACACGGGGAATAG
*L*. *pentosus* IG1	FR874854.1_Crispr_1	289548	289944	396	7	GGGATCACCCCCGTATACACGGGGAATACA
	FR874854.1_Crispr_2	299897	300172	275	5	CTATTCCCCGTGTATACGGGGGTGATCCT
	FR874854.1_Crispr_3	585210	585665	455	8	CTGTTCCCCGTGTATGCGGGGGTGATCC
	FR874854.1_Crispr_4	788797	788983	186	4	GTTGTACCACCGCCATCGCCGGGG
	FR874854.1_Crispr_5*	790101	790233	132	3	GTTGTACCACCGCCATCGCCGGGG
	FR874854.1_Crispr_6	920329	920758	429	7	TCTTGACCTTATTGATTTAATGTCCTTCTGAAAC
	FR874854.1_Crispr_7*	1504524	1504670	146	2	GGATTGATGTAAACAGGTGCACGTGACTTAGCTACGGATTG
*L*. *pentosus* FL0421	tmp_1_Crispr_1*	221528	221664	136	2	AAACAGGTGTACGTGACTTAGCTACGGATTG
	tmp_1_Crispr_2	466666	467162	496	8	GTTCTAAACCTGTTTGATATGACTACTATTCAAGAC
*L*. *plantarum* CF_001296095	NZ_CP012343_2	2563734	2564693	959	15	GTCTTGAATAGTAGTCATATCAAACAGGTTTAGAAC
*L*. *plantarum* ZJ316	NC_020229_1	359930	360361	431	7	GTCTTGAATAGTAGTCATATCAAACAGGTTTAGAAC
*L*. *plantarum* GCF_001296095	NZ_CP012343_2	2563734	2564693		15	GTCTTGAATAGTAGTCATATCAAACAGGTTTAGAAC
*L*. *plantarum* GCF_001715615	NZ_CP015308_2	1823736	1824036		5	GTTCTAAACCTGTTTGATATGACTACTATTCAAGAC
*L*. *plantarum* GCF_001660025	NZ_CP015857_1	2311451	2312014		9	GTTCTAAACCTGTTTGATATGACTACTATTCAAGAC
*L*. *plantarum* GCF_001659745	NZ_CP015966_1	2416755	2417252		8	GTTCTAAACCTGTTTGATATGACTACTATTCAAGAC
*L*. *plantarum* subsp. *plantarum* GCF_001272315	NZ_CM003439_1	2774673	2775303	630	10	GTCTTGAATAGTAGTCATATCAAACAGGTTTAGAAC
*L*. *paraplantarum* GCF_001443645	NZ_CP013130_1	302519	303280	761	12	GGTCTTGACCTTATTGATTTAATGTCCTTCTGAAAC
	NZ_CP013130_2	1344198	1344530	332	6	GGATCACCCCCGCATACACGGGGAACAG
	NZ_CP013130_3	1349145	1349416	271	5	GGATCACCCCCGTATGCACGGGGAATAG
	NZ_CP013130_4	1351689	1352203	514	9	GGATCACCCCCGTATACACGGGGAATAG
	NZ_CP013130_5*	2726056	2726234	178	3	GTCACCTTAGAACAATTCTGAAA
*L*. *brevis* GCF_001676805	NZ_CP015398_1	79605	80762	1157	18	GTTCTTAACCCTATTGATTTACCAAGATTCTAAAGC
	NZ_CP015398_2	229570	229735	165	3	GGATCACCCCCACACCTGTGGGGAATAC
	NZ_CP015398_3	391217	391302	85	2	GTATTCCCCACATGTGTGGGGGTGA
	NZ_CP015398_4	1416352	1416623	271	5	GTATTCCCCACGGGTGTGGGGGTGATCC
*L*. *brevis* ATCC 367	NC_008497_1	944684	945017	333	6	AGGATCACCCCCACATGTGTGGGGAATAC
	NC_008497_2	2249734	2250005	271	5	GGATCACCCCCACACCTGTGGGGAATAC

*: Questionable CRISPR array.

**: The same DR consensus sequences are indicated by the same color and their reverse complement was underlined.

Each CRISPR array comprised of short spacer sequences that were fragments of foreign DNA, either derived from the phage or plasmid, incorporated into the host between degenerate repeats (DR consensus). The number of confirmed CRISPR arrays was similar in both *L*. *pentosus* strains (MP-10 and KCA1); however, the number of repeats and spacers, the CRISPR length, and the DR consensus sequence were different, although two identical repeats were found in both *L*. *pentosus* strains (MP-10 and KCA1) (Table 1). Comparison of CRISPR arrays of *L*. *pentosus* MP-10 and phylogenetically related lactobacilli, such as *L*. *plantarum*, *L*. *paraplantarum* and *L*. *brevis* (available in CRISPRs database), showed that one DR consensus (5´-GTCTTGAATAGTAGTCATATCAAACAGGTTTAGAAC-3´) or its reverse complement was shared by all *L*. *pentosus* and *L*. *plantarum* strains except *L*. *pentosus* IG1 (Table 1). Such DR consensus could be considered as a more conserved repeat signature in *L*. *plantarum* group.

The number of spacers ranged from four in CR5 to eleven in CR6 identified within the six confirmed CRISPR arrays with lengths ranging from 29 to 51 bp (40 bp average length) (Table 2). The search of protospacer was done using CRISPR Target program to localize the DNA target acquired by horizontal gene transfer, and the results revealed the presence of protospacers related to plasmids and phages. These protospacers were located within genes encoding structural viral protein (such as tail-fiber protein) or bacterial enzymes such as thioredoxin reductase, short-chain dehydrogenase, excinuclease ABC subunit A and FMN-dependent oxidoreductase, nitrilotriacetate monooxygenase family protein, et al. (Table 2). Furthermore, the protospacers were also identified within genes of unknown function and in intergenic regions (Table 2).

**Table 2:** Characteristics of spacers from CRISPR arrays in *Lactobacillus pentosus* MP-10 genome as revealed by CRISPRTarget program.

CRISPR array	Spacer sequence (5´-3´)	Protospacer characteristics					
		Origin of DNA	Position	Strand	Score	Accession number	Gene (GenBank)
**CR1**	AAAATCATTTGTAAAGTTCAATGGCTTGTT	*Haematospirillum jordaniae* H5569 Plasmid unnamed 2	262527..262506	-	20	NZ_CP014527.1	Non coding
	GACGCTAACGATCGCCCAACTAAGGTATGGTTACC	X	X	X	X	X	X
	CGCTTGCATGGTACAATAGGAACATGGCAGCGGA	X	X	X	X	X	X
	CGGATGGTCTGCACCTGCGCT	X	X	X	X	X	X
	GGAACGATGGGGAAATAAAGGTTCGCGCCAAGAG	X	X	X	X	X	X
	TATCAGGATGCCCTAAAGACTGCTA	X	X	X	X	X	X
	TTTAAATTCTCCTTTATCTCTTATCGTTTT	*Borrelia miyamotoi* FR64b Plasmid_07	15826..15799	-	20	NZ_CP004224.1	Non coding
		*Clostridium taeniosporum* 1/k Plasmid pCt3	119290..119311	+	20	NZ_CP017256.1	Thioredoxin reductase
	TTGCTGTTAAGCTAACTGGCGACATGAGCATTCCC	X	X	X	X	X	X
	ATATTTCCGTTCAAACAACGTAACT	X	X	X	X	X	X
	CGAGCCAAACAAAATTTCGATGTTCAGCAA	X	X	X	X	X	X
CR2*	ACATCAATCCGTAGCTAAGTCACGTGCACCTGTTTACATCAATCCATAGCAAAACCAACGTGCACTTGTTTTCAA	X	X	X	X	X	X
**CR3**	TCATCTAGTAGATGAATTTGATTGTGGAAATAGG	*Buchnera aphidicola* str. Ua (Uroleucon ambrosiae) Plasmid pLeu	1180..1206	+	21	NC_017261.1	Non coding
	CAAGTGTTCTGCGAAGAAGCGCTGACAAAAGCCA	*Pseudomonas* Phage phiPSA1	7572..7597	+	20	KJ507100	Tail fiber protein
	AAAGTCTAAATTTCCGTTCGAATCTTTAAACCA	X	X	X	X	X	X
	ATGACAAGACCAACGATGCGAAGTCCAATGTAA	X	X	X	X	X	X
	ATGCACGAATCGGCGGAACATCCGCCGACAACA	X	X	X	X	X	X
	AAAATATGTTGACCGGTATCGGGCGGGTAACAA	X	X	X	X	X	X
	GAGCGTTCCTTTTTGGCACGGGATTGTTATTCG	Ensifer adhaerens Casida A Plasmid pCasidaAA	246999..247027	+	21	NZ_CP015881.1	Non coding
	TACAATGTACTTGTAGATAAGGAAAGGAAGTTA						
	CGCCTTCGCGGTCACGAAAACCGCGATGATGAT	*Shinella* sp. HZN7 Plasmid pShin-01	346033..346060	+	22	NZ_CP015737.1	TonB-dependent receptor
		*Burkholderia phymatum* STM815 Plasmid pBPHY01	1636942..1636911	-	22	NC_010625.1	Short-chain dehydrogenase
		*Novosphingobium resinovorum* SA1 Plasmid pSA2	269117..269088	-	20	NZ_CP017077.1	Excinuclease ABC subunit A
		*Sinorhizobium* sp. RAC02 Plasmid pBSY16_1	1283345..1283370	+	20	NZ_CP016452.1	FMN-dependent oxidoreductase, nitrilotriacetate monooxygenase family protein
		*Escherichia coli* PMV-1 pHUSEC411like plasmid	11436..11413	-	20	NC_022371.1	Non coding
		*Burkholderia phenoliruptrix* BR3459a Plasmid pSYMBR3459	597126..597105	-	20	NC_018696.1	Non coding
		*Ralstonia eutropha* JMP134 Megaplasmid	24652..24681	+	20	NC_007336.1	Excinuclease ABC, A subunit
CR4*	GGTTGCAGCGGTGCTCGTTGCTTGA	X	X	X	X	X	X
**CR5**	TATGAGTGGCTGATTGTAAACAATGAATTAGAGG	*Acinetobacter baumannii* MDR-TJ Plasmid pABTJ1	72649..72622	-	20	NC_017848.1	Hypothetical protein
		*Acinetobacter baumannii* BJAB07104 Plasmid p1BJAB07104	3093..3066	-	20	NC_021727.1	Hypothetical protein
		*Acinetobacter baumannii* BJAB0868 Plasmid p2BJAB0868	3093..3066	-	20	NC_021731.1	Hypothetical protein
	CCTGTCGTCATTGATGTAACGGATGGTACCGAG	X	X	X	X	X	X
	CGAACCGGGTACTTGTGTTATTAGGGCTTGTTG	X	X	X	X	X	X
	CAAATCTTCTGAATCACTAATCGCTGAAGCTGA	*Bacillus* Phage Eldridge	35750..35781	+	20	KU253712	Hypothetical protein
**CR6**	GTAAAAAACTTTATCCACTCCATGCGCTCCTTG	X	X	X	X	X	X
	GATTGAGAATCTGCAAAACCCGTTAAGCCCTTA	X	X	X	X	X	X
	CCTAATCCAGTCAAACTCATGCCGTTTCGAACA	X	X	X	X	X	X
	AAATACTTATCTTTTGAGACAGCCAACCACATG	*Moraxella* Phage Mcat17	53007..53034	+	20	KR093641	Non coding
	CATTGATATGGTGGGTTTTTGTTTTGCCAAAAAG	X	X	X	X	X	X
	TGAAGTTTAAGCTGCAGCGCGAAGCTATTGGTA	X	X	X	X	X	X
	CGTTGGCACTTAACGCCGCTATTGGCCTGATGA	*Ensifer adhaerens* OV14 Plasmid pOV14b	1574834..1574861	+	20	NZ_CP007239.1	NADH:ubiquinone oxidoreductase
	GTCAAGCGTTCAGCTTTGTCGACACCGACGTTA	X	X	X	X	X	X
	CAACTTAACCCTTACCAATTGGTAAGGGTTTTA	X	X	X	X	X	X
	TATCGTAGTTAGTCAAATGCATGACGCGATTCG	X	X	X	X	X	X
	GCCGTTAATTTCGTAATAAAATCATCGTAACCA	*Leuconostoc gelidum* subsp. *gasicomitatum* KG16-1 Plasmid: III	21115..21141	+	21	NZ_LN890333.1	Conjugal transfer protein
**CR7**	GTTCCAAATATAGGAATGTCAATCGGTCACTAAG	X	X	X	X	X	X
	GAATGTGAAGCTGCCCGTATATCGCATCATTAAG	X	X	X	X	X	X
	CGATGTTCTTGTAATACCAAGCTTGTTCTCCCGGG	X	X	X	X	X	X
	AGTGCTTTGGTATCATACCGATCAGCGACTTTGGG	X	X	X	X	X	X
	TGTGAACGCGCAAACGTCTGAATACAGCAAGTAG	X	X	X	X	X	X
	GAGTATTTCCCGCCCGTGGCTGAGGCATTTTGAG	X	X	X	X	X	X
	AATAGTGCAAACTTCACCAAAATGGCAACGCAGG	X	X	X	X	X	X
	TCGCCGCTAGTACCAGTAGCAATCCAATATCCAGG	*Enterococcus faecalis* Plasmid pBEE99	1574..1547	-	20	NC_013533	Non coding
**CR8**	TGAACCGTTGGATGAGTTGTTGTCATCCACATCATCATCACTAGGCGTCGT	X	X	X	X	X	X
	TGTAGTCGTACCAGTGCCGCCACCATTGATGTTGTCGCCAGT	*Geminocystis* sp. NIES-3709 Plasmid pGM3709_05	9880..9908	+	21	NZ_AP014826.1	Hypothetical protein
		*Rhizobium* sp. LPU83 Plasmid pLPU83d	1927939..1927909	-	21	NZ_HG916855.1	Hypothetical protein
		*Oscillatoria nigro-viridis* PCC 7112 Plasmid pOSC7112.02	27040..27007	-	20	NC_019730.1	Cobyrinic acid a,c-diamide synthase
		*Pseudomonas* Phage 17A	16695..16720	+	20	LN889995	Non coding
		*Pseudomonas* Phage vB_PaeM_PAO1_Ab29	38037..38008	-	20	LN610588	Hypothetical protein
		*Pseudomonas* Phage S12-1	29421..29392	-	20	LC102730	Phage protein
		*Pseudomonas* Phage vB_PaeM_CEB_DP1	30502..30473	-	20	KR869157	Putative structural protein
		*Pseudomonas* Phage phiKTN6	29954..29925	-	20	KP340288	Structural protein
		*Pseudomonas* Phage phiKT28	30552..30523	-	20	KP340287	Structural protein
		*Pseudomonas* Phage NH-4	30503..30474	-	20	JN254800	Hypothetical protein
		*Pseudomonas* Phage SN	30731..30702	-	20	FM887021	Structural protein
		*Pseudomonas* Phage LMA2	30502..30473	-	20	FM201282	Putative structural protein
		*Pseudomonas* Phage KPP12	29436..29407	-	20	AB560486	Putative structural protein
	GCTGCCACCACCATTGTTACCGTTGTCACCAGT	*Klebsiella variicola* DX120E Plasmid pKV2	50267..50292	+	20	NZ_CP009276.1	Non coding
		*Burkholderia caribensis* MBA4 Plasmid	1469077..1469048	-	20	NZ_CP012748.1	Hypothetical protein
		*Lactobacillus plantarum* Bacteriophage LP65	62235..62260	+	20	AY682195	Non coding
CR9*	GGTTGCAGCGGTGCTCGTTGCTTGA	X	X	X	X	X	X

X: No results obtained by CRISPRTarget program. HP: Hypothetical protein. ND: Not determined.

Given that the spacers were usually added at one side of the CRISPR system, the chronological record of the viruses and plasmids (protospacers), which invaded *L*. *pentosus* MP-10 or its ancestors, could be detected by searching for the spacers with BLAST (Basic Local Alignment Search Tool). For example in CR1, we suggested that the primary invasion was accomplished by *Haematospirillum jordaniae* H5569 Plasmid unnamed 2, then by other short sequences followed by *Borrelia miyamotoi* FR64b Plasmid_07, and *Clostridium taeniosporum* 1/k Plasmid pCt3 (Table 2). On the other hand, multiple targets were observed for all confirmed CRISPR spacers of *L*. *pentosus* MP-10 except for CR7 (Table 2). This suggests that *L*. *pentosus* MP-10 could target many diverse viruses and plasmids. As such, they could possess an efficient defense mechanism against different pathogens, not only in food systems, but also in intestinal tract—thus reinforcing their probiotic capacity.

**Fig 4:**
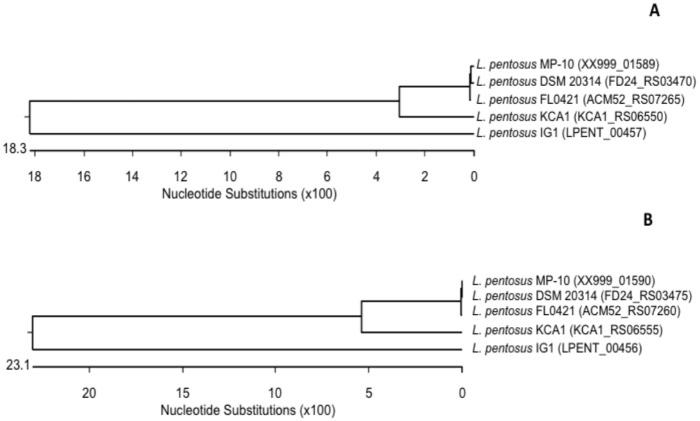
Phylogenetic relationships of *L*. *pentosus* inferred from the alignment of the CRISPR-associated proteins encoding genes [*cse1* (A) and *cse2* (B)]. The sequences were aligned and the most parsimonious phylogenetic trees were constructed using the CLUSTAL W of Lasergene program, version 14 (MegAlign 14, Inc., Madison, WI, USA). The scale below indicates the number of nucleotide substitutions. Accession numbers are indicated in parentheses.

Regarding the CRISPR-associated protein involved in sequence-specific recognition and cleavage of target DNA complementary to the spacer, according to the classification suggested by Makarova et al. [26], three major types of the CRISPR-Cas systems were differentiated (Types I, II and III). However, in the present study both signature genes for the Type I (*cas3*) and Type II (*cas9*) systems were detected in *L*. *pentosus* MP-10 genome (S1 Table, Fig 3B). CRISPR1 and CRISPR2 consisted of three Type-II-C and eight Type-I genes, respectively (Fig 3B), and they were closely associated with the palindromic repeat/spacer units (Fig 3A). CRISPR1 operon consisted of only three genes (*cas1*, *cas2* and *cas9*), which were similar to those of *Streptococcus thermophilus* (S1 Table) and adjacent to the CR1 array (Fig 3A). A comparison of *L*. *pentosus* MP-10 and *L*. *pentosus* KCA1 revealed that CRISPR1 of *L*. *pentosus* KCA1 contained one more gene encoding a protein involved in adaptation (the *csn2* gene) [27]; while CRISPR1 of *L*. *pentosus* KCA1 belonged to Type II-A, CRISPR1 of *L*. *pentosus* MP-10 belonged to Type II-C lacking this fourth gene (Fig 3B). Regarding CRISPR2 of *L*. *pentosus* MP-10, this operon consisted of eight genes: the coding genes for CRISPR-associated endonucleases Cas1 and Cas2 (*ygbT* and *ygbF* genes); the CRISPR system Cascade subunit CasC (*casC* gene); and the CRISPR system Cascade subunit Cas5 (XX999_01592 gene ID of *L*. *pentosus* MP-10), which were similar to *Escherichia coli*, the Cas3 nuclease/helicase (*cas3* gene) in *Streptococcus thermophilus*, the CRISPR-associated endoribonuclease Cse3 in *Thermus thermophilus* and two genes unique for *L*. *pentosus* MP-10 (XX999_01589 gene ID, or *cse1_Lpe* gene, and XX999_01590 gene ID, or *cse2_Lpe* gene) (S1 Table). Among the eight genes of CRISPR2, five of them were shared by both *L*. *pentosus* strains (MP-10 and KCA1): *cas1*, *cas2*, *cas3*, *casC*, *cas5* and *cse3* (Fig 3B); however, both unique genes for *L*. *pentosus* MP-10 (XX999_01589 gene ID, or *cse1_Lpe* gene, and XX999_01590 gene ID, or *cse2_Lpe* gene) corresponded to CRISPR-associated protein (KCA1_RS06550) and *cse2*/*casB* (KCA1_RS06555) in *L*. *pentosus* KCA1. Alignment of these genes revealed that the *cse1-Lpe* gene from *L*. *pentosus* MP-10 showed high similarity to the CRISPR-associated protein from *L*. *pentosus* DSM 20314 and *L*. *pentosus* FL0421 (99.8% identity) and also with *L*. *pentosus* KCA1 (94.2%). However, it showed only 71.6% identity with *cse1* gene sequence from *L*. *pentosus* IG1, which formed a separate lineage from the other cluster representing the four lactobacilli (Fig 4A). On the other hand, the *cse2-Lpe* gene from *L*. *pentosus* MP-10 was identical to the *cse2* gene from *L*. *pentosus* DSM 20314 and *L*. *pentosus* FL0421 (100% identity) and highly similar to *cse2/casB* gene from *L*. *pentosus* KCA1 (90.2% identity); however, *L*. *pentosus* IG1 formed a different lineage (67.3% identity) from the main cluster of other lactobacilli (Fig 4B). It is noteworthy to highlight that the CRISPR genes found in *L*. *pentosus* MP-10 were more highly similar to those of *L*. *pentosus* DSM 20314 (isolated from corn silage), *L*. *pentosus* FL0421 (isolated from temperate deciduous-forest biome soil), and *L*. *pentosus* KCA1 (isolated from the vagina), than *L*. *pentosus* IG1 isolated from fermented olives. These data provided new insight into the evolution of bacterial resistance against mobile elements in *Lactobacillus* spp., which highlight their interconnection between different ecosystems; thus *L*. *pentosus* MP-10 possess multiple CRISPR elements of various nature, which are (again) of great relevance for the application of this bacterium, not only as a promising probiotic, but also as starter culture at industrial scale.

### Detection of mobile genetic elements in *Lactobacillus pentosus* MP-10 genome

**Fig 5:**
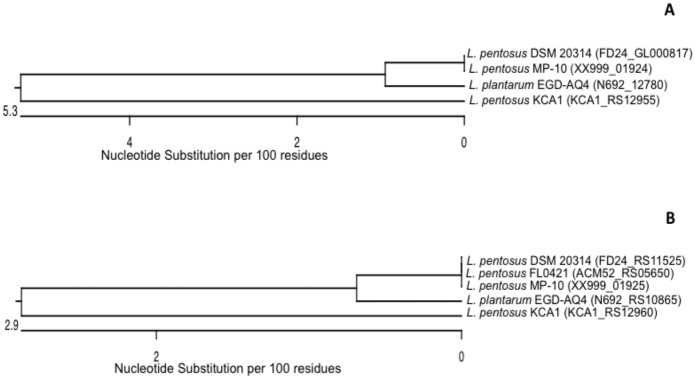
Phylogenetic relationships of *L*. *pentosus* and *L*. *plantarum* inferred from the alignment of the transposase encoding genes. The sequences were aligned and the most parsimonious phylogenetic trees were constructed using the CLUSTAL W of Lasergene program, version 14 (MegAlign 14, Inc., Madison, WI, USA). The scale below indicates the number of nucleotide substitutions. Accession numbers are indicated in parentheses.

Bacterial genome of *L*. *pentosus* MP-10 included 29 transposase, four putative transposon Tn*552* DNA-invertase bin3 (four different genes of the same family) located on plasmids (pLPE-2, pLPE-3, pLPE-4 and pLPE-5), and one transposase repressor (IS2 repressor *TnpA*) coding gene. The transposases represented nine different families, with three of them appearing in multiple copies ranging from three to six (Table 3). Furthermore, they were highly represented by the DDE superfamily: 17 transposase DDE domain proteins (five different genes), which appeared in 5–7 copies as a result of replication events. Other transposases were represented by three transposases (three different genes), three transposases of the mutator family (three different genes), two putative transposases (two different genes, with a single gene unique to *L*. *pentosus* MP-10), two transposase IS200 like proteins (two different genes, with one gene unique to *L*. *pentosus* MP-10), one transposase from transposon Tn*916* and one IS2 transposase *TnpB* coding gene. Similarity of *L*. *pentosus* MP-10 transposase genes was shown to transposases from other *Lactobacillus* spp.: mainly *L*. *plantarum*, *L*. *fermentum*, and *L*. *brevis* (Table 3). The number of transposase genes present in *L*. *pentosus* MP-10 (29 genes) was higher than other lactobacilli strains such as *L*. *pentosus* KCA1 (25 genes) [20], *L*. *acidophilus* NCFM (18 genes) [28], *L*. *pentosus* DSM 20314 (14 genes) and *L*. *pentosus* IG1 (five genes) which suggested that insertion element-mediated genome diversification was more frequent in the *L*. *pentosus* MP-10 environment (Table 3). Furthermore, BLASTx analysis of transposase-unique genes, predicted in *L*. *pentosus* MP-10, revealed similarly encoded proteins in other lactobacilli, and the result further showed that the encoded transposase of *L*. *pentosus* MP-10 had similarity with transposase proteins of *L*. *pentosus* KCA1, *L*. *pentosus* DSM 20314 and *L*. *pentosus* FL0421 (Fig 5). ClustalW alignment of *XX999_01924* putative transposase and other transposase genes showed 100% identity to transposase gene from *L*. *pentosus* DSM 20314 (Fig 5A); however, it was more similar to *L*. *plantarum* EGD-AQ4 (98.2% identity) than to *L*. *pentosus* KCA1 (90.3% identity) transposases (Fig 5A). Regarding the transposase IS200-like protein encoding gene (*XX999_01925*), alignment with ClustalW with other related genes showed 100% identity to *L*. *pentosus* FL0421 and *L*. *pentosus* DSM 20314 (Fig 5B); however, similarly we observed less homology to the encoding gene for the transposase-IS200-like protein from *L*. *pentosus* KCA1 (94.9% identity) than to *L*. *plantarum* EGD-AQ4 (98.6% identity) (Fig 5B).

**Table 3:** Characterization of transposase and transposon elements predicted in *Lactobacillus pentosus* MP-10 genome.

Gene ID	Gene	Position	Strand	Gen length (bp)	Protein description	Protein family	Similarity to transposase in Lactobacillus*
XX999_00032^§^	*bin3_1*	24835–25416	-	582	Putative transposon Tn*552* DNA-invertase bin3	UniProtKB:P20384	98% identity transposase in *L*. *paracollinoides* TMW 1.1995 plasmid pL11995-6
XX999_00061^£^	*XX999_00061*	6507–6758	-	252	Transposase	Pfam:PF01527.14	100% identity transposase in *L*. *lindneri* TMW 1.481
XX999_00069^£^	*XX999_00069*	14032–14613	-	582	Transposase, Mutator family	Pfam:PF00872.12	99% identity transposase in *L*. *fermentum* 47–7
XX999_00071^£^	*bin3_2*	17298–17972	-	675	Putative transposon Tn*552* DNA-invertase bin3	UniProtKB:P20384	99% identity transposase in *L*. *fermentum* IFO 3956
XX999_00112	*XX999_00112*	22929–23432	-	504	Transposase DDE domain protein	Pfam:PF01609.15	99% identity transposase in *L*. *plantarum* LY-78
XX999_00245	*XX999_00245*	157564–158067	-	504	Transposase DDE domain protein	Pfam:PF01609.15	99% identity transposase in *L*. *plantarum* LY-78
XX999_00336	*XX999_00336*	260525–261202	+	678	IS2 repressor *TnpA*	CLUSTERS:PRK09413	100% identity transposase in *L*. *plantarum* AY01
XX999_00337	*XX999_00337*	261379–262110	+	732	IS2 transposase *TnpB*	CLUSTERS:PRK09409	100% identity transposase in *L*. *plantarum* MF1298 plasmid unnamed7
XX999_00400	*XX999_00400*	331304–331807	-	504	Transposase DDE domain protein	Pfam:PF01609.15	99% identity transposase in *L*. *plantarum* LY-78
XX999_00407	*XX999_00407*	334530–334901	+	372	Transposase DDE domain protein	Pfam:PF01609.15	99% identity transposase in *L*. *plantarum* subsp. *plantarum* TS12
XX999_00611	*XX999_00611*	565747–566250	-	504	Transposase DDE domain protein	Pfam:PF01609.15	99% identity transposase in *L*. *plantarum* LY-78
XX999_00680	*Int-Tn*	637701–638858	-	1158	Transposase from transposon Tn*916*	UniProtKB:P22886	97% identity transposase in *L*. *plantarum* LZ206
XX999_01017	*XX999_01017*	992606–992803	+	198	Transposase	Pfam:PF01527.14	100% identity transposase in *L*. *pentosus* IG1
XX999_01502	*XX999_01502*	1519616–1519912	+	297	Transposase DDE domain protein	Pfam:PF01609.15	99% identity transposase in *L*. *plantarum* C410L1 plasmid unnamed1
XX999_01619	*XX999_01619*	1648272–1648775	+	504	Transposase DDE domain protein	Pfam:PF01609.15	99% identity transposase in *L*.. *plantarum* LY-78
XX999_01924	*XX999_01924*	1973033–1974301	-	1269	Putative transposase	Pfam:PF01385.13	-
XX999_01925	*XX999_01925*	1974399–1974839	+	441	Transposase IS200 like protein	Pfam:PF01797.10	-
XX999_02663	*XX999_02663*	2747991–2749130	-	1140	Putative transposase DNA-binding domain protein	Pfam:PF07282.5	75% identity transposase in *L*. *brevis* BSO 464 plasmid pLb464-1
XX999_02664	*XX999_02664*	2749111–2749563	-	453	Transposase IS200 like protein	Pfam:PF01797.10	80% identity transposase in *L*. *brevis* BSO 464 plasmid pLb464-1
XX999_02834	*XX999_02834*	2935214–2935510	+	297	Transposase DDE domain protein	Pfam:PF01609.15	99% identity transposase in *L*. *plantarum* LZ227 plasmid LZ227p2
XX999_02924	*XX999_02924*	3033618–3033914	+	297	Transposase DDE domain protein	Pfam:PF01609.15	99% identity transposase in *L*. *plantarum* C410L1 plasmid unnamed1
XX999_02993	*XX999_02993*	3117440–3117943	+	504	Transposase DDE domain protein	Pfam:PF01609.15	99% identity transposase in *L*. *plantarum* LY-78
XX999_03221	*XX999_03221*	3359214–3359585	+	372	Transposase DDE domain protein	Pfam:PF01609.15	99% identity transposase in *L*. *plantarum* subsp. *plantarum* TS12
XX999_03439	*XX999_03439*	3608820–3609191	-	372	Transposase DDE domain protein	Pfam:PF01609.15	99% identity transposase in *L*. *plantarum* subsp. *plantarum* TS12
XX999_03498	*XX999_03498*	3674577–3674948	+	372	Transposase DDE domain protein	Pfam:PF01609.15	99% identity transposase in *L*. *plantarum* subsp. *plantarum* TS12
XX999_03585^#^	*XX999_03585*	24998–25501	-	504	Transposase DDE domain protein	Pfam:PF01609.15	99% identity transposase in *L*. *plantarum* subsp. *plantarum* P-8 plasmid LBPp7
XX999_03604^#^	*bin3_3*	40077–40709	+	633	Putative transposon Tn*552* DNA-invertase bin3	UniProtKB:P20384	100% identity transposase in *L*. *backii* TMW 1.1992 plasmid pL11992-1
XX999_03610^#^	*XX999_03610*	45885–46475	-	591	Transposase, Mutator family	Pfam:PF00872.12	100% identity transposase in *L*. *backii* TMW 1.1992 plasmid pL11992-1
XX999_03614^¥^	*XX999_03614*	4535–5902	-	1368	Transposase DDE domain protein	Pfam:PF01609.15	-
XX999_03618^¥^	*XX999_03618*	9187–9690	+	504	Transposase DDE domain protein	Pfam:PF01609.15	100% identity transposase in *L*. *plantarum* BM4 plasmid pBM2
XX999_03623^¥^	*XX999_03623*	13862–15037	+	1176	Transposase, Mutator family	Pfam:PF00872.12	99% identity transposase in *L*. *acidipiscis* ACA-DC 1533
XX999_03627^¥^	*XX999_03627*	17186–17482	+	297	Transposase DDE domain protein	Pfam:PF01609.15	99% identity transposase in *L*. *plantarum* C410L1 plasmid unnamed1
XX999_03633^¥^	*bin3_4*	22401–23033	-	633	Putative transposon Tn*552* DNA-invertase bin3	UniProtKB:P20384	99% identity transposase in *L*. *plantarum* ZJ316 plasmid pLP-ZJ103

*: The best hit was indicated.

^§^: sequences of pLPE-4 plasmid;

^£^: sequences of pLPE-3 plasmid;

^#^: sequences of pLPE-5 plasmid;

^¥^: sequences of pLPE-2 plasmid.

On the other hand, screening for prophage DNA within *L*. *pentosus* MP-10 genome, using bioinformatic tools such as PHAST, determined the presence of five temperate phage regions. Two regions were intact (Regions 2 and 5, score > 90), the other two were questionable (Regions 1 and 4, score 70–90), and the last one was incomplete (region 3, score < 70) (Fig 3A, Table 4). The complete prophage regions of *L*. *pentosus* MP-10 chromosome were identified as *Lactobacillus* phage Sha1 (region 2; GC content, 40.35%; region length, 39.2 kb) [29] and *Oenococcus* phage phi 9805 (region 5; GC content, 42.21%; region length, 51.7 kb) [30]. The questionable prophage regions corresponded to *Streptococcus pyogenes* phage 315.2 (region 1; GC content, 42.18%; region length, 15.4 kb) [29] and *Listeria* phage B025 (region 4; GC content, 42.96%; region length, 20.9 kb) [31]. The incomplete prophage region was identified as *Lactobacillus* phage Sha1 (region 3; GC content, 42.61; region length, 26.7 kb) [29]. The occurrence of prophage DNA within bacterial genomes is common; over 40 *Lactobacillus* prophages have been reported [32] and their presence highlights the genetic diversity and fitness of the *Lactobacillus* genome. In our case, the presence of prophages may confer selective advantage to the cell, promoting its survivability and its resistance to other infecting phages.

**Table 4:** Description of prophage regions detected in *L*. *pentosus* MP-10 genome by using the PHAST bioinformatic tool.

Region	Region length	Completeness*	Score	Region position	Most common phage	GC%	Total proteins
1	15.4 kb	Questionable	80	39530–54980	PHAGE_Strept_315.2_NC_004585(3)	42.18	24
2	39.2 kb	Intact	150	637535–676738	PHAGE_Lactob_Sha1_NC_019489(27)	40.35	49
3	26.7 kb	Incomplete	40	1405091–1431841	PHAGE_Lactob_Sha1_NC_019489(7)	42.61	25
4	20.9 kb	Questionable	80	1437486–1458462	PHAGE_Lister_B025_NC_009812(8)	42.96	21
5	51.7 kb	Intact	120	2437004–2488736	PHAGE_Oenoco_phi9805_NC_023559 (16)	42.21	57

*: Intact (score > 90), Questionable (score 70–90), Incomplete (score < 70).

S2 Table shows the proteins encoded by the five prophage regions predicted by PHAST tool in *L*. *pentosus* MP-10 genome. The complete prophages corresponded to regions 2 and 5 encoded 49 and 57 proteins, respectively (Table 4) and were homologous to *Lactobacillus* phage Sha1 isolated from traditional Korean fermented food “kimchi” [29] and *Oenococcus* phage phi 9805 from red wine [30]. Those data suggest that different species colonizing different ecosystems may share the same prophages and their architecture due to the interconnection between different habitats via lateral genetic exchange [33].

Each prophage region of *L*. *pentosus* MP-10 genome showed the presence of an integrase: one integrase in each complete prophage (region 2 and 5), two integrases in incomplete prophage (region 3), and a single integrase in the questionable prophage (region 1) (S2 Table); also phage attachment sites (attL and attR) (in regions 1, 2, 3 and 5) were found to be potentially involved in the integration of prophage regions in host chromosome. However, screening of the whole genome (outside prophage regions) of *L*. *pentosus* MP-10 for phage integrases as markers for mobile DNA elements, such as prophages, determined the presence of fifteen integrase core domain proteins not adjacent to the prophage-like region, thus we deduce that they were not involved in prophage mobility (data not shown). However, lysis genes (endolysin and holin) detected in prophage regions may be used by *L*. *pentosus* MP-10 in their own ecological niche or could be used in the food industry to eliminate undesirable bacteria during fermentation, particularly in cheese making to accelerate ripening. However, studies concerning the application of *L*. *pentosus* MP-10 in several fermentations should be studied in depth.

### *In silico* analysis of safety properties of *L*. *pentosus* MP-10

To generate further insights into the food-safety aspects of *L*. *pentosus* MP-10, we surveyed the genes related with antibiotic resistance and virulence factors in their genome.

#### Antibiotic resistance

**Fig 6:**
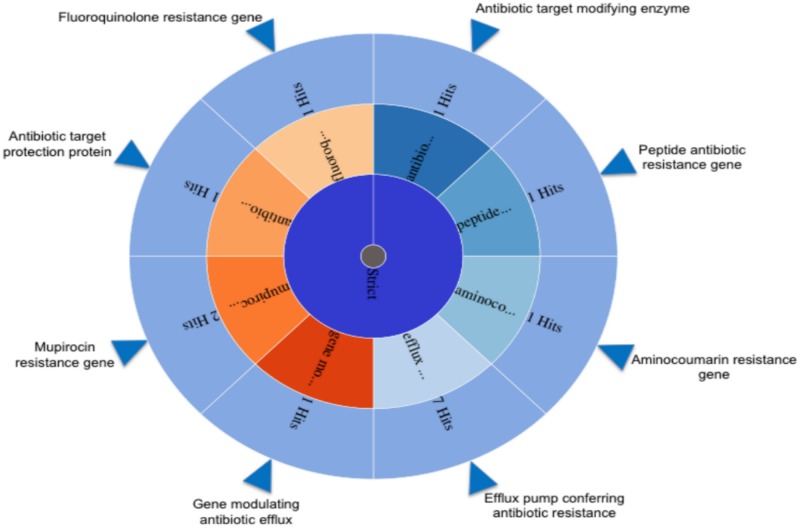
Screening of the whole genome of *Lactobacillus pentosus* MP-10 by using the perfect and strict algorithms in the Resistance Gene Identifier (RGI) with overall resistance in the center, resistance classes in the middle, and individual resistance genes on the outer (open reading frames).

Firstly, a BLAST search was conducted for each annotated element of *L*. *pentosus* MP-10 genome sequence against the antibiotic resistance genes database (CARD). The search predicted the presence of several genes involved in antibiotic resistance although their identity to known resistance genes were low (< 90%), thus we could not suggest that the genes in *L*. *pentosus* MP-10 genome were homologous to the described genes (data not shown). To predict the complete resistome from *L*. *pentosus* MP-10 genome, including resistance genes and mutations conferring antibiotic resistance, we used the Resistance Gene Identifier (RGI) tool available in the recent updated CARD database [34], which used archive’s curated AMR (antimicrobial resistance) detection models. Here, we detected strict hits, which were defined as being within the similarity cut-offs of the individual AMR detection models and represented likely homologs of AMR genes according to Jia et al. [34]. The RGI revealed that *L*. *pentosus* MP-10 chromosome contained specific resistance genes for different antibiotics: aminocoumarin (*alaS*, an alanyl-tRNA synthetase gene, 1 hit), fluoroquinolone (*mfd* gene, 1 hit) and mupirocin (*ileS* or isoleucyl-tRNA synthetase gene, 2 hits), as well as genes coding for efflux pump proteins conferring resistance to multiple antibiotics (Fig 6, S3 Table). Among them, we found LmrB and LmrD multidrug efflux pumps that confer resistance to lincosamides in *Bacillus subtilis*, and *Streptomyces lincolnensis* and *Lactococcus lactis*, respectively [35–36]; the regulator of ArlR efflux-pump that binds to the *norA* promoter to activate its expression [37]; and the multidrug efflux pump EmeA from *Enterococcus faecalis* conferring resistance to several antimicrobial agents (S3 Table). Previous phenotypic analysis of antibiotic susceptibility of *L*. *pentosus* MP-10 [38] revealed that this strain showed resistance to cefuroxime, ciprofloxacin, teicoplanin, trimethoprim, trimethoprim/sulfamethoxazole and vancomycin. However, *L*. *pentosus* MP-10 was sensitive to clindamycin [38], thus *lmrB* and *lmrD* genes coding for multidrug efflux pumps were not involved in clindamycin resistance.

On the other hand, a loose algorithm, which works outside of the detection model cut-offs to provide detection of new, emergent threats and more distant homologs of AMR genes [34], was also used; S4 Table shows the results. Considering the previous results of antibiotic resistance phenotypic screening [38], we can suggest that resistance to cefuroxime, ciprofloxacin, teicoplanin, trimethoprim, trimethoprim/sulfamethoxazole and vancomycin may be mediated by new genes responsible (not determined up to date) for the intrinsic resistance; however, further studies are required to confirm this hypothesis.

Regarding the possibility of acquired resistance by horizontal gene transfer, ResFinder did not detect any acquired antibiotic resistance genes for aminoglycoside, beta-lactam, colistin, fluoroquinolone, fosfomycin, fusidic acid, MLS-series (macrolide, lincosamide and streptogramin B), nitroimidazole, oxazolidinone, phenicol, rifampicin, sulphonamide, trimethoprim, tetracycline and glycopeptide (data not shown).

In summary, *in silico* analysis of antibiotic resistance in *L*. *pentosus* MP-10 showed the absence of acquired antibiotic resistance genes, and the resistome was mostly represented by efflux-pump resistance genes responsible of the intrinsic resistance exhibited by this strain.

#### Virulence

Regarding virulence, the BLAST searches against a virulence gene database (PHAST) revealed the presence of 14 coding genes for P1, P2a and P2b prophage proteins, an alanine racemase and a DNA-binding ferritin-like protein similar to *L*. *plantarum* WCFS1 (>90% identity; Table 5). As such, *Lb*. *pentosus* MP-10 chromosome contained mostly P2b prophage elements, which were located in the predicted questionable prophage region (Region 1, Fig 3A; PHAGE_Strept_315.2_NC_004585(3)], Table 4), and included: DNA packaging genes (encoding small and large terminase, portal protein), head-tail genes (head-to-tail joining), helicase and DNA replication gene (Table 5). These results were in accordance of those reported in S2 Table for Region 1. Furthermore, several proteins of unknown functions of P2b (proteins 10 and 21) prophage from *Lb*. *plantarum* WCFS1 were also detected (Table 5); however, van Hemert et al. [39] showed that prophage P2b protein 21 was involved in modulating peripheral blood mononuclear cell (PBMC) cytokine interleukin 10 (IL-10) and IL-12 production, which might be responsible for the stimulation of anti- or pro-inflammatory immune responses in the gut. Comparing P2b prophage region of *Lb*. *pentosus* MP-10 and *Lb*. *plantarum* WCFS1, we observed a strong synteny between prophage regionss from the two distinct species of *Lactobacillus*, despite the comparison being done with proteins with >90% identity (Table 5). In this case, nine homologous proteins were shared, although each species occupies a different ecological niches: human saliva and olives [16, 40], respectively. Similar results were reported by Zhang et al. [41] for other lactobacilli.

**Table 5:** Characterization of virulence determinants predicted in *Lactobacillus pentosus* MP-10 genome against the MvirDB database of virulence factors.

Gene ID	Identity (%)	Query length	Subject length	E-value	Protein Description	Organism	Accession
XX999_00145	92.08	101	101	1E-60	Prophage P2b protein 21	*L*. *plantarum* WCFS1	CCC79635.1
XX999_00131	92.48	266	266	0.0	Prophage P2b protein 7, DNA replication	*L*. *plantarum* WCFS1	CCC79647.1
XX999_00596	92.53	375	375	0.0	Alanine racemase	*L*. *plantarum* WCFS1	UniProtKB—O08
XX999_02401	92.68	127	126	9e-83	Prophage P2a protein 24, endodeoxyribonuclease	*L*. *plantarum* WCFS1	CCC79612.1
XX999_00135	93.65	63	63	2e-36	Prophage P2b protein 10	*L*. *plantarum* WCFS1	CCC79644.1
XX999_00137	93.80	129	129	2e-88	Prophage P2b protein 12, endonuclease	*L*. *plantarum* WCFS1	CCC79642.1
XX999_02409	95.05	101	101	7e-69	Prophage P2a protein 12	*L*. *plantarum* WCFS1	YP_004890137.1
XX999_02999	95.48	155	155	5e-108	DNA-binding ferritin-like protein, DPS family	*L*. *plantarum* WCFS1	CCC80168.1
XX999_01408	95.83	170	169	2e-117	Prophage P2a protein 16	*L*. *plantarum* WCFS1	CCC79619.1
XX999_02421	96.00	138	138	6e-87	Prophage P1 protein 7	*L*. *plantarum* WCFS1	CCC78108.1
XX999_00141	96.72	368	366	0.0	Prophage P2b protein 17, portal protein	*L*. *plantarum* WCFS1	CCC79639.1
XX999_00138	96.82	157	157	1e-111	Prophage P2b protein 14, terminase small subunit	*L*. *plantarum* WCFS1	CCC79641.1
XX999_00132	96.98	464	464	0.0	Prophage P2b protein 8, helicase	*L*. *plantarum* WCFS1	CCC79646.1
XX999_00139	97.53	567	567	0.0	Prophage P2b protein 15, terminase large subunit	*L*. *plantarum* WCFS1	CCC79640.1
XX999_00143	97.70	89	89	2e-56	Prophage P2b protein 19, head-to-tail joining	*L*. *plantarum* WCFS1	CCC79637.1
XX999_02397	99.34	152	153	3e-111	Prophage P1 protein 33, phage transcription regulator	*L*. *plantarum* WCFS1	CCC78134.1

### Concluding notes

The new annotated genome sequence of *L*. *pentosus* MP-10 is currently considered the largest genome among lactobacilli; their additional genes may reflect the microorganism’s ecological flexibility and adaptability. *In silico* analysis of the genome identified a CRISPR (clustered regularly interspaced short palindromic repeats)/cas (CRISPR-associated protein genes) system involved in bacterial resistance against mobile elements, which consisted of six arrays (4–12 repeats) and eleven predicted *cas* genes (CRISPR1 and CRISPR2 consisted of three TypeII-C and eight TypeI-E genes) with high similarity to *L*. *pentosus* KCA1. Bioinformatic evidence of *L*. *pentosus* MP-10 did not reveal any acquired antibiotic resistance genes, and most inherent resistance genes were antibiotic efflux genes. No virulence factors were found. Thus, we can suggest that *L*. *pentosus* MP-10 could be considered safe for food processing, and high their adaptation potential could facilitate their application as a probiotic and starter culture in industrial processes.

## Materials and methods

### Genome sequence of *L*. *pentosus* MP-10

The complete genome sequence of *L*. *pentosus* MP-10 was obtained by using PacBio RS II technology [17] and deposited at the EMBL Nucleotide Sequence Database (accession numbers FLYG01000001 to FLYG01000006). The assembled genome sequences were annotated at Lifesequencing S.L. (Valencia, Spain) using the Prokka annotation pipeline, version 1.11 [42]. This involved predicting tRNA, rRNA, and mRNA genes and signal peptides in the sequences using Aragorn, RNAmmer, Prodigal, and SignalP, respectively, [43–45].

To evaluate the alignment and the synteny of genes between the *L*. *pentosus* MP-10, *L*. *pentosus* KCA1 and *L*. *pentosus* IG1 genome data sets, comparison was done by using Mauve algorithm in Lasergene's MegAlign Pro software (Lasergene 14).

### Genomic analysis of mobile genetic elements and safety aspects of *Lactobacillus pentosus* MP-10

The annotated genome sequence of *L*. *pentosus* MP-10 was screened for the presence of CRISPR (Clustered Regularly Interspaced Short Palindromic Repeats) loci and the mobile genetic elements (i.e., conjugative plasmid, transposase, transposon, IS elements and prophage). Furthermore, we used the CRISPR finder tool (available in the CRISPRs web server; http://crispr.i2bc.paris-saclay.fr/Server/) to identify CRISPRs and extract the repeated and unique sequences in the *L*. *pentosus* MP-10 genome. The localization of CRISPR RNAs targets was done by using CRISPR Target program (http://bioanalysis.otago.ac.nz/CRISPRTarget/crispr_analysis.html). For prophage region search and annotation, we screened chromosomal DNA of *L*. *pentosus* MP-10 against a phage finding tool (PHAST, PHAge Search Tool) considered as an accurate or slightly more accurate than most available phage finding tools, with sensitivity of 85.4% and positive predictive value of 94.2% [46].

The predicted CDSs were annotated by using BLAST (Basic Local Alignment Search Tool) against the CARD (Comprehensive Antibiotic Resistance Database) and the MvirDB (a microbial database of protein toxins, virulence factors and antibiotic resistance genes for bio-defence applications) databases for antibiotic resistance and virulence factor screening (last version downloaded on January, 2017), respectively, with the associated GO (Gene Ontology) terms obtained by using Swiss-Prot database. Furthermore, the Resistance Gene Identifier (RGI) software (as part of CARD tools) was used for prediction of *L*. *pentosus* MP-10 resistome from protein or nucleotide data based on homology and SNP (Single Nucleotide Polymorphism) models, based on the CARD′s curated AMR (antimicrobial resistance) detection models. Moreover, the ResFinder (acquired antimicrobial Resistance gene Finder) software version 2.1 (https://cge.cbs.dtu.dk//services/ResFinder/) was used for screening of acquired antibiotic resistance genes [47] with selected %ID threshold of 90.00% and Selected minimum length of 60% (last accessed in January, 2017).

## Supporting information

S1 Fig COG distributions in *Lactobacillus pentosus* MP-10. (PDF)

S1 Table Characterization of CRISPR associated proteins predicted in *Lactobacillus pentosus* MP-10 genome. (DOC)

S2 Table Characteristics of prophage regions in *Lactobacillus pentosus* MP-10 genome according to the PHAST bioinformatic toolkit. (DOC)

S3 Table RGI results of AMR genes detected in *Lactobacillus pentosus* MP-10 genome. (DOC)

S4 Table AMR detected in *Lactobacillus pentosus* MP-10 genome by using hits with weak “loose” similarity in RGI software. (DOC)
