# Supplementary figures and images for: In silico genomic insights into aspects of food safety and defense mechanisms of a potentially probiotic Lactobacillus pentosus MP-10 isolated from brines of naturally fermented Aloreña green table olives
Source: PLoS One. 2017 Jun 26;12(6):e0176801. doi: 10.1371/journal.pone.0176801 (PMC5484467; doi:10.1371/journal.pone.0176801)

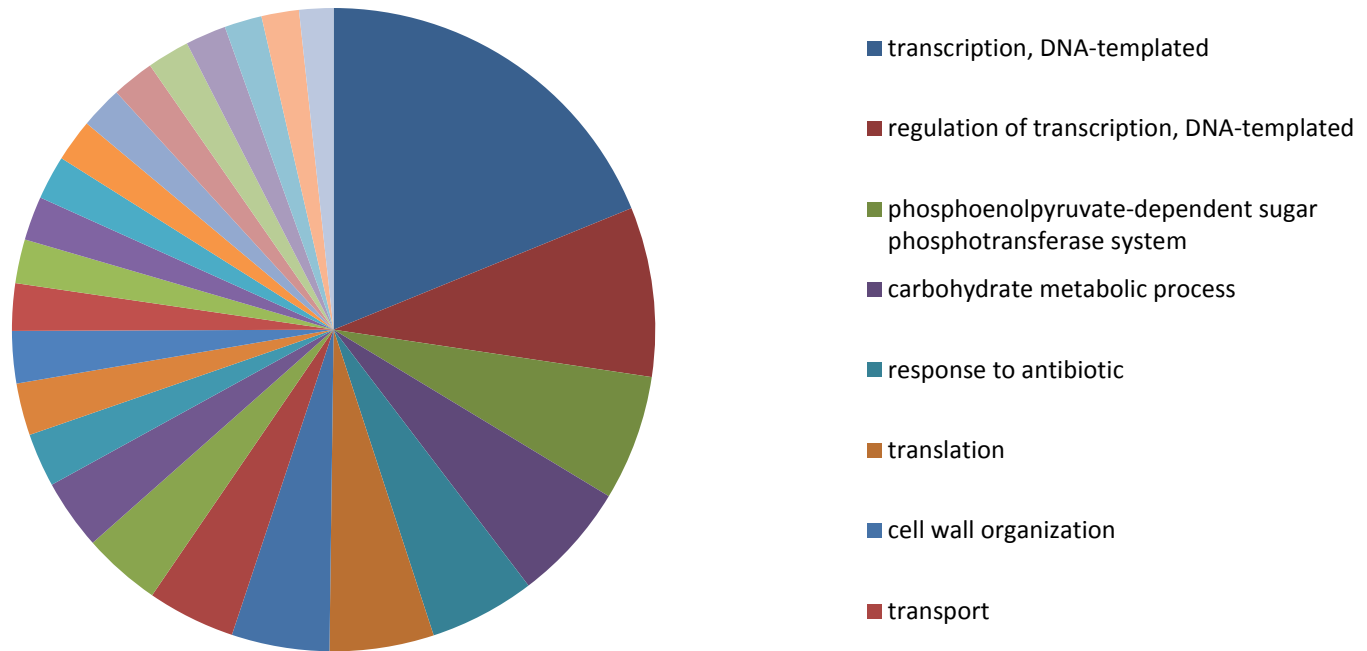

**Figure S1**

**Abriouel et al.**

Supplement: S1 Fig — (PDF) [file pone.0176801.s001.pdf]
