# Supplementary material for: In silico genomic insights into aspects of food safety and defense mechanisms of a potentially probiotic Lactobacillus pentosus MP-10 isolated from brines of naturally fermented Aloreña green table olives
Source: PLoS One. 2017 Jun 26;12(6):e0176801. doi: 10.1371/journal.pone.0176801 (PMC5484467; doi:10.1371/journal.pone.0176801)
Supplement: S1 Table — (DOC) [file pone.0176801.s002.doc]

| **Gene ID** | **Gene** | **Position** | **Gen length (bp)** | **Protein description** | **UniProt Reference Clusters (UniRef)** | **Predicted functions** |
| --- | --- | --- | --- | --- | --- | --- |
| XX999_00242  XX999_00243  XX999_00244  XX999_01586  XX999_01587  XX999_01588  XX999_01589  XX999_01590  XX999_01591  XX999_01592  XX999_01593 | *cas9*  *cas1*  *cas2*  *ygbT*  *ygbF*  *cas3*  *cse1_Lpe**  *cse2_Lpe**  *casC*  *casD*  *cse3* | 152032-156108  156302-157207  157185-157490  1611954-1612907  1612904-1613803  1615061-1617808  1617813-1619564  1619554-1620165  1620165-1621244  1621225-1621950  1621950-1622618 | 4077  906  306  954  900  2748  1752  612  1080  726  669 | CRISPR-associated endonuclease Cas9  CRISPR-associated endonuclease Cas1  CRISPR-associated endoribonuclease Cas2  CRISPR-associated endonuclease Cas1  CRISPR-associated endoribonuclease Cas2  CRISPR-associated nuclease/helicase Cas3  CRISPR-associated protein Cse1 (CRISPR_cse1)  CRISPR-associated protein Cse2 (CRISPR_cse2)  CRISPR system Cascade subunit CasC  CRISPR-associated protein (Cas_Cas5)  CRISPR-associated endoribonuclease Cse3 | UniRef100:G3ECR1  UniRef100:G3ECR2  UniRef100:G3ECR  UniRef100:Q46896  UniRef100:P45956  UniRef100:F2XG53  -  -  UniRef100:Q46899  UniRef100:Q46898  UniRef100:Q53WG9 | DNA and RNA binding; endonuclease activity; maintenance of CRISPR repeat elements; metal ion binding; defense response to virus  DNA binding; endonuclease activity; maintenance of CRISPR repeat elements; metal ion binding; defense response to virus  Endonuclease activity; maintenance of CRISPR repeat elements; metal ion binding; defense response to virus  DNA binding; cytoplasm; DNA repair; cellular response to DNA damage stimulus; crossover junction endodeoxyribonuclease activity; 5'-flap endonuclease activity; maintenance of CRISPR repeat elements; metal ion binding; defense response to virus  Endonuclease activity; maintenance of CRISPR repeat elements; defense response to virus  Nucleic acid binding; helicase activity; deoxyribonuclease activity; ATP binding; DNA metabolic process; metal ion binding  -  -  RNA binding; protein complex; defense response to virus  RNA binding; protein complex; defense response to virus  RNA binding; endonuclease activity; defense response to virus |

**Table S1.** Characterization of CRISPR associated proteins predicted in *Lactobacillus pentosus* MP-10 genome.

*: New genes founds in this study.
