# Supplementary material for: In silico genomic insights into aspects of food safety and defense mechanisms of a potentially probiotic Lactobacillus pentosus MP-10 isolated from brines of naturally fermented Aloreña green table olives
Source: PLoS One. 2017 Jun 26;12(6):e0176801. doi: 10.1371/journal.pone.0176801 (PMC5484467; doi:10.1371/journal.pone.0176801)
Supplement: S2 Table — (DOC) [file pone.0176801.s003.doc]

**Table S2.** Characteristics of prophage regions in *Lactobacillus pentosus* MP-10 genome according to PHAST bioinformatic tool.

| **Region position** | **BLAST HIT*** | **E-Value** |
| --- | --- | --- |
|
| **Region 1 (24 CDS):**  complement(39530..39973)  40173..40373  40462..40683  40703..40816  40829..40847  complement(40900..41043)  complement(41124..42278)  complement(42336..43022)  43154..43336  10 43605..43835  11 43849..44649  12 44649..46043  13 46059..46193  14 46190..46609  15 46633..46935  16 46935..47126  17 47113..47454  18 47447..47836  19 48450..48923  20 48920..50623  21 50577..50777  22 50778..51884  23 51874..53454  24 53567..53836  25 53995..54375  26 54980..54998 | PHAGE_Lactob_PLE3_NC_031125: hypothetical protein; PP_00032; phage(gi100030)  PHAGE_Lactoc_bIL312_NC_002671: Csp; PP_00033; phage(gi13095918)  Hypothetical protein zj316_0256 [*Lactobacillus plantarum* ZJ316]. gi|448819779|ref|YP_007412941.1|; PP_00034  hypothetical; PP_00035  attL TATGATGGGCAGTCAGGGG  hypothetical; PP_00036  PROPHAGE_Oceano_HTE831: integrase; PP_00037; phage(gi23097608)  PHAGE_Lactob_KC5a_NC_007924: putative transcriptional regulator; PP_00038; phage(gi90592588)  prophage P2b protein 4 [*Lactobacillus plantarum* WCFS1]. gi|380033173|ref|YP_004890164.1|; PP_00039  hypothetical protein JDM1_0034 [*Lactobacillus plantarum* JDM1]. gi|254555203|ref|YP_003061620.1|; PP_00040  PHAGE_Lactoc_bIL310_NC_002669: hypothetical protein; PP_00041; phage(gi13095885)  PHAGE_Staphy_2638A_NC_007051: ORF003; PP_00042; phage(gi66395453)  hypothetical; PP_00043  prophage P3 protein 12 [*Lactobacillus plantarum* 16]. gi|513841805|ref|YP_008121777.1|; PP_00044  hypothetical; PP_00045  prophage P2b protein 10 [*Lactobacillus plantarum* WCFS1]. gi|380033167|ref|YP_004890158.1|; PP_00046  PHAGE_Lactob_Sha1_NC_019489: phage head-tail adaptor; PP_00047; phage(gi418489808)  PHAGE_Staphy_phiPV83_NC_002486: phi PVL ORF 63 homologue; PP_00048; phage(gi9635713)  PHAGE_Entero_IME_EFm5_NC_028826: terminase small subunit; PP_00049; phage(gi971746949)  PHAGE_Lactob_LF1_NC_019486: bacteriophage terminase large subunit; PP_00050; phage(gi418489385)  hypothetical protein Lp16_1955 [*Lactobacillus plantarum* 16]. gi|513841799|ref|YP_008121771.1|; PP_00051  PHAGE_Entero_EFAP_1_NC_012419: portal protein; PP_00052; phage(gi225626395)  PHAGE_Entero_IME_EFm5_NC_028826: capsid protein; PP_00053; phage(gi971746944)  PHAGE_Entero_EFAP_1_NC_012419: head-tail joining protein; PP_00054; phage(gi225626391)  PHAGE_Lactob_PL_1_NC_022757: transcriptional regulator; PP_00055; phage(gi557308042)  attR TATGATGGGCAGTCAGGGG | 2e-13  2e-23  7e-33  0.0  0.0  0.0  2e-71  3e-12  2e-14  5e-30  2e-18  2e-70  0.0  1e-74  0.0  3e-26  3e-11  7e-20  2e-15  4e-116  5e-30  1e-47  1e-41  4e-06  9e-07  0.0 |
| **Region 2 (49 CDS):**  637535..637550  complement(637701..638858)  complement(639773..639854)  complement(640315..640497)  complement(640524..640620)  complement(640626..640699)  complement(640714..640799)  complement(640990..641190)  complement(641251..641472)  complement(641651..641983)  642241..642462  642477..643211  643223..643432  643445..643576  complement(643548..643874)  43942..644220  644355..644594  644606..644806  645091..645261  645452..645568  645667..646470  646500..647339  647562..647795  647952..648128  648121..648258  648395..648628  648874..649302  650031..650150  650134..650304  650315..650782  650981..651439  651442..653340  653330..653524  653527..654720  654695..655441  655456..656688  656768..657103  657440..657847  657847..658227  658243..658890  658963..659337  659382..659567  659609..663247  663485..664108  664184..665956  666020..668374  668389..671175  671724..671885  671869..672228  672240..673412  673412..673675  673688..674218  674944..675075  675096..675599  676738..676753 | attL AATCTTCTTGGCCGCA  PHAGE_Lactob_Lj965_NC_005355: putative integrase; PP_00601; phage(gi41179218)  tRNA  hypothetical protein LPST_C1709 [*Lactobacillus plantarum* subsp. *plantarum* ST-III]. gi|308180891|ref|YP_003925019.1|; PP_00602  tRNA  tRNA  tRNA  PHAGE_Lactob_Sha1_NC_019489: hypothetical protein; PP_00603; phage(gi418489829)  PHAGE_Lactob_PLE2_NC_031036: tail length tape-measure protein 1; PP_00604; phage(gi100028)  PHAGE_Lactob_PLE2_NC_031036: hypothetical protein; PP_00605; phage(gi100029)  Hypothetical protein zj316_2078 [*Lactobacillus plantarum* ZJ316]. gi|448821601|ref|YP_007414763.1|; PP_00606  PHAGE_Lactob_PLE2_NC_031036: major tail protein; PP_00607; phage(gi100031)  PHAGE_Lactob_phiadh_NC_000896: hypothetical protein; PP_00608; phage(gi9633008)  PHAGE_Lactob_Sha1_NC_019489: hypothetical protein; PP_00609; phage(gi418489834)  PHAGE_Lactob_Sha1_NC_019489: RNA polymerase III; PP_00610; phage(gi418489835)  hypothetical protein JDM1_0478 [*Lactobacillus plantarum* JDM1]. gi|254555647|ref|YP_003062064.1|; PP_00611  PHAGE_Lactob_Sha1_NC_019489: hypothetical protein; PP_00612; phage(gi418489839)  PHAGE_Lactob_Sha1_NC_019489: hypothetical protein; PP_00613; phage(gi418489840)  hypothetical protein LPST_C1694 [*Lactobacillus plantarum* subsp. *plantarum* ST-III]. gi|308180876|ref|YP_003925004.1|; PP_00614  hypothetical; PP_00615  PHAGE_Lactob_LF1_NC_019486: DNA replication protein; PP_00616; phage(gi418489431)  PHAGE_Lactob_iA2_NC_028830: DNA replication protein; PP_00617; phage(gi971747669)  PHAGE_Lactob_Sha1_NC_019489: hypothetical protein; PP_00618; phage(gi418489790)  PHAGE_Lactob_Sha1_NC_019489: hypothetical protein; PP_00619; phage(gi418489795)  hypothetical; PP_00620  PHAGE_Entero_phiFL2A_NC_013643: hypothetical protein; PP_00621; phage(gi281416518)  PHAGE_Lactob_Sha1_NC_019489: phage transcriptional activator RinA; PP_00622; phage(gi418489798)  Putative prophage protein [*Lactobacillus plantarum* ZJ316]. gi|448821584|ref|YP_007414746.1|; PP_00623  PHAGE_Lactob_Sha1_NC_019489: HNH nuclease; PP_00624; phage(gi418489799)  PHAGE_Lactob_Sha1_NC_019489: restriction endonuclease; PP_00625; phage(gi418489800)  PHAGE_Lactob_Sha1_NC_019489: P27 family phage terminase small subunit; PP_00626; phage(gi418489801)  PHAGE_Lactob_Sha1_NC_019489: phage terminase-like protein large subunit; PP_00627; phage(gi418489802)  PHAGE_Lactob_Sha1_NC_019489: phage head-tail joining protein; PP_00628; phage(gi418489803)  PHAGE_Lactob_Sha1_NC_019489: phage portal protein; PP_00629; phage(gi418489804)  PHAGE_Lactob_Sha1_NC_019489: protease subunit of ATP-dependent Clp protease; PP_00630; phage(gi418489805)  PHAGE_Lactob_Sha1_NC_019489: HK97 family phage major capsid protein; PP_00631; phage(gi418489806)  PHAGE_Lactob_Sha1_NC_019489: phage protein DNA packaging protein; PP_00632; phage(gi418489807)  PHAGE_Strept_DT1_NC_002072: putative tail component protein; PP_00633; phage(gi9632427)  PHAGE_Strept_Sfi21_NC_000872: putative tail component protein; PP_00634; phage(gi9632947)  PHAGE_Lactob_phiAT3_NC_005893: putative major tail protein; PP_00635; phage(gi48697269)  hypothetical protein JDM1_0994 [*Lactobacillus plantarum* JDM1]. gi|254556161|ref|YP_003062578.1|; PP_00636  PHAGE_Lactob_Sha1_NC_019489: hypothetical protein; PP_00637; phage(gi418489813)  PHAGE_Lactob_Sha1_NC_019489: minor tail protein; PP_00638; phage(gi418489814)  PHAGE_Lactob_Sha1_NC_019489: minor tail protein; PP_00639; phage(gi418489814)  PHAGE_Lactob_Sha1_NC_019489: minor structural protein gp75-like protein; PP_00640; phage(gi418489815)  PHAGE_Lactob_Sha1_NC_019489: minor structural protein gp89-like protein; PP_00641; phage(gi418489816)  PHAGE_Lactob_Sha1_NC_019489: tail fiber; PP_00642; phage(gi418489817)  PHAGE_Lactob_Sha1_NC_019489: hypothetical protein; PP_00643; phage(gi418489819)  PHAGE_Lactob_Sha1_NC_019489: prophage Lp2 protein 53-like protein; PP_00644; phage(gi418489820)  PHAGE_Lactob_Sha1_NC_019489: endolysin; PP_00645; phage(gi418489821)  PHAGE_Lactob_Sha1_NC_019489: phage-related holin; PP_00646; phage(gi418489822)  PHAGE_Lactob_Sha1_NC_019489: hypothetical protein; PP_00647; phage(gi418489823)  hypothetical; PP_00648  PHAGE_Lactob_Lb338_1_NC_012530: hypothetical protein; PP_00649; phage(gi226377705)  attR AATCTTCTTGGCCGCA | 0.0  1e-44  0.0  4e-16  0.0  0.0  0.0  4e-31  2e-13  4e-30  5e-33  2e-42  5e-05  1e-17  1e-57  5e-33  6e-37  1e-27  1e-20  0.0  7e-45  9e-51  3e-32  2e-13  0.0  5e-07  3e-56  1e-15  2e-24  1e-85  1e-82  0.0  9e-31  0.0  5e-127  7e-79  5e-47  1e-27  2e-15  1e-23  5e-58  2e-05  2e-171  4e-29  0.0  0.0  1e-71  6e-21  7e-49  0.0  2e-41  1e-80  0.0  4e-08  0.0 |
| **Region 3 (25 CDS):**    1405091..1405103  1405267..1417245  complement(1417655..1418317)  complement(1418314..1419312)  complement(1419383..1419907)  complement(1420563..1421186)  complement(1421295..1421687)  complement(1421954..1422385)  complement(1422395..1422775)  1423082..1423228  1423241..1424050  1424047..1424250  1424250..1424588  1424702..1424941  1425407..1425739  1425751..1425921  1426175..1426306  1426299..1426811  1426814..1427461  1427458..1427862  1427877..1428569  1428616..1429383  1429383..1430168  1430304..1430483  1430923..1431081  1431408..1431818  1431841..1431853 | attL GATGAAAAATGAG  PHAGE_Sphing_PAU_NC_019521: gp103; PP_01318; phage(gi435844606)  PHAGE_Lactob_Sha1_NC_019489: phage integrase; PP_01319; phage(gi418489824)  PHAGE_Staphy_StauST398_4_NC_023499: putative transposase; PP_01320; phage(gi588498272)  PHAGE_Lactob_Sha1_NC_019489: phage integrase; PP_01321; phage(gi418489824)  Hypothetical protein zj316_1370 [*Lactobacillus plantarum* ZJ316]. gi|448820893|ref|YP_007414055.1|; PP_01322  hypothetical; PP_01323  PHAGE_Lactob_phig1e_NC_004305: hypothetical protein; PP_01324; phage(gi23455774)  PHAGE_Lactob_phig1e_NC_004305: repressor; PP_01325; phage(gi23455773)  PHAGE_Bacill_BCJA1c_NC_006557: cro; PP_01326; phage(gi56694875)  PHAGE_Lactob_phiadh_NC_000896: hypothetical protein; PP_01327; phage(gi9633007)  Hypothetical protein zj316_1375 [*Lactobacillus plantarum* ZJ316]. gi|448820898|ref|YP_007414060.1|; PP_01328  PHAGE_Lactob_Sha1_NC_019489: hypothetical protein; PP_01329; phage(gi418489836)  PHAGE_Lactob_Sha1_NC_019489: hypothetical protein; PP_01330; phage(gi418489839)  hypothetical; PP_01331  hypothetical protein LPST_C1694 [*Lactobacillus plantarum* subsp. *plantarum* ST-III]. gi|308180876|ref|YP_003925004.1|; PP_01332  prophage P2a protein 15 [*Lactobacillus plantarum* WCFS1]. gi|380033143|ref|YP_004890134.1|; PP_01333  PHAGE_Lactob_phig1e_NC_004305: hypothetical protein; PP_01334; phage(gi254854755)  PHAGE_Lactob_LBR48_NC_027990: hypothetical protein; PP_01335; phage(gi937456720)  PHAGE_Lactob_LBR48_NC_027990: putative single strand binding protein; PP_01336; phage(gi937456721)  PHAGE_Lactob_Sha1_NC_019489: hypothetical protein; PP_01337; phage(gi418489846)  PHAGE_Bacill_vB_BhaS_171_NC_030904: hypothetical protein; PP_01338; phage(gi100052)  PHAGE_Lactob_Sha1_NC_019489: DNA replication protein; PP_01339; phage(gi418489789)  PHAGE_Lactob_Sha1_NC_019489: hypothetical protein; PP_01340; phage(gi418489790)  PHAGE_Lactob_Sha1_NC_019489: hypothetical protein; PP_01341; phage(gi418489791)  PHAGE_Staphy_Ipla5_NC_018281: YopX family protein; PP_01342; phage(gi399528952)  attR GATGAAAAATGAG | 0.0  1e-41  3e-55  7e-55  4e-30  1e-98  0.0  2e-31  4e-21  6e-11  3e-60  2e-27  5e-32  6e-37  0.0  6e-10  2e-17  4e-58  8e-66  5e-46  2e-119  8e-43  4e-146  1e-25  2e-08  1e-12  0.0 |
| **Region 4 (21 CDS):**  1437486..1437773  1437770..1439449  1439468..1440610  1440597..1441319  1441342..1442292  1442344..1442514  1442653..1442958  1442939..1443328  1443325..1443732  1443729..1444151  1444166..1444777  1444869..1445183  1445447..1449991  1449995..1450816  1450836..1455731  1455749..1456240  1456242..1456688  1456693..1457076  1457079..1457273  1457273..1457557  1457557..1458462 | PHAGE_Lister_B025_NC_009812: gp1; PP_01352; phage(gi157325218)  PHAGE_Lister_B025_NC_009812: putative terminase large subunit; PP_01353; phage(gi157325219)  PHAGE_Staphy_phi5967PVL_NC_019921: phage portal protein; PP_01354; phage(gi431810270)  PHAGE_Lister_B025_NC_009812: gp4; PP_01355; phage(gi157325221)  PHAGE_Lister_B025_NC_009812: Cps; PP_01356; phage(gi157325222)  PHAGE_Staphy_phiBU01_NC_026016: capsid protein; PP_01357; phage(gi744692789)  PHAGE_Lister_B025_NC_009812: gp7; PP_01358; phage(gi157325224)  PHAGE_Lister_B025_NC_009812: gp8; PP_01359; phage(gi157325226)  PHAGE_Lister_B025_NC_009812: gp9; PP_01360; phage(gi157325225)  PHAGE_Lister_B025_NC_009812: gp10; PP_01361; phage(gi157325227)  PHAGE_Lister_B025_NC_009812: Tsh; PP_01362; phage(gi157325228)  hypothetical protein JDM1_0510 [*Lactobacillus plantarum* JDM1]. gi|254555679|ref|YP_003062096.1|; PP_01363  PHAGE_Lactob_LF1_NC_019486: phage tail tape measure protein; PP_01364; phage(gi418489397)  PHAGE_Entero_phiFL1A_NC_013646: tail protein; PP_01365; phage(gi281416378)  PHAGE_Lactob_ATCC8014_NC_019916: prophage tail super family protein; PP_01366; phage(gi431809813)  PHAGE_Strept_phi3396_NC_009018: hypothetical protein; PP_01367; phage(gi126011113)  hypothetical protein JDM1_0516 [*Lactobacillus plantarum* JDM1]. gi|254555685|ref|YP_003062102.1|; PP_01368  PHAGE_Lactob_LfeSau_NC_029068: hypothetical protein; PP_01369; phage(gi985757745)  PHAGE_Lactob_phijl1_NC_006936: hypothetical protein; PP_01370; phage(gi62327114)  PHAGE_Lactob_phig1e_NC_004305: holin; PP_01371; phage(gi23455817)  PHAGE_Entero_EF62phi_NC_017732: endolysin type Endo-N-acetylmuramidase; PP_01372; phage(gi384519788) | 1e-14  5e-145  3e-95  3e-54  1e-73  2e-10  1e-10  2e-21  6e-13  9e-15  5e-19  5e-47  0.0  3e-31  3e-76  4e-10  8e-58  2e-32  2e-05  1e-06  1e-40 |
| **Region 5 (57 CDS):**  complement(2437004..2438890)  complement(2438890..2440620)  2440392..2440403  complement(2440841..2441239)  2441473..2442333  complement(2442531..2442603)  2442738..2442944  2442993..2443065  complement(2444113..2445087)  complement(2445216..2445593)  complement(2445605..2445868)  complement(2445868..2447040)  complement(2447168..2447602)  complement(2447605..2447991)  complement(2448061..2453379)  complement(2453394..2453726)  complement(2453770..2459601)  complement(2459617..2459820)  complement(2459988..2460386)  complement(2460486..2460956)  complement(2460971..2461336)  complement(2461336..2461887)  complement(2461889..2462236)  complement(2462236..2462568)  complement(2462580..2462756)  complement(2462769..2463791)  complement(2463811..2464158)  complement(2464173..2464850)  complement(2465028..2466248)  complement(2466265..2466387)  complement(2466395..2466580)  complement(2466621..2468129)  complement(2468141..2469379)  complement(2469369..2469896)  complement(2469936..2470199)  complement(2470420..2470536)  complement(2470517..2471011)  complement(2471468..2471539)  complement(2471609..2471681)  complement(2471804..2472265)  complement(2472347..2472580)  complement(2472612..2472746)  complement(2473163..2473291)  complement(2473543..2474043)  complement(2474057..2474962)  complement(2475043..2476017)  complement(2476029..2476559)  complement(2476580..2476693)  complement(2476857..2476970)  complement(2476982..2477494)  complement(2477561..2477866)  complement(2478073..2478369)  complement(2478369..2478575)  2478948..2479826  2479864..2480178  2481218..2481580  2481592..2482008  2482081..2482449  2482479..2483180  2483214..2483305  2483426..2484598  complement(2484737..2484886)  2485665..2485750  2487246..2487257  2487272..2487355  2487370..2487443  2487449..2487519  2487615..2488736 | PHAGE_Bacill_SPbeta_NC_001884: ABC transporter; PP_02296; phage(gi9630145)  PHAGE_Bacill_SPbeta_NC_001884: ABC transporter; PP_02297; phage(gi9630145)  attL CCGCCAAGAAGA  PHAGE_Staphy_StB20_like_NC_028821: hypothetical protein; PP_02298; phage(gi971746471)  PHAGE_Bacill_BCP8_2_NC_027355: putative polysaccharide deacetylase; PP_02299; phage(gi849252969)  tRNA  prophage P2b protein 24 [*Lactobacillus plantarum* WCFS1]. gi|380033157|ref|YP_004890148.1|; PP_02300  tRNA  PHAGE_Escher_phiV10_NC_007804: O-acetyltransferase; PP_02301; phage(gi89152472)  PHAGE_Staphy_phiMR11_NC_010147: putative holin protein; PP_02302; phage(gi162290172)  PHAGE_Lactob_Sha1_NC_019489: phage-related holin; PP_02303; phage(gi418489822)  PHAGE_Lactob_PLE3_NC_031125: tail fiber protein; PP_02304; phage(gi100023)  PHAGE_Lactob_Ldl1_NC_026609: hypothetical protein; PP_02305; phage(gi764162089)  PHAGE_Strept_315.6_NC_004589: hypothetical protein; PP_02306; phage(gi28876446)  PHAGE_Lactob_Lj965_NC_005355: hypothetical protein; PP_02307; phage(gi41179262)  PHAGE_Lactob_Lj965_NC_005355: hypothetical protein; PP_02308; phage(gi41179261)  PHAGE_Lactob_Lj965_NC_005355: putative putative minor tail protein; PP_02309; phage(gi41179260)  PHAGE_Lactob_Lj965_NC_005355: hypothetical protein; PP_02310; phage(gi41179259)  PHAGE_Lactob_Lj965_NC_005355: hypothetical protein; PP_02311; phage(gi41179250)  PHAGE_Lactob_Lj965_NC_005355: putative major tail protein; PP_02312; phage(gi41179249)  PHAGE_Lactob_Lj965_NC_005355: hypothetical protein; PP_02313; phage(gi41179248)  PHAGE_Lactob_Lj965_NC_005355: hypothetical protein; PP_02314; phage(gi41179258)  PHAGE_Lactob_Lj965_NC_005355: hypothetical protein; PP_02315; phage(gi41179247)  PHAGE_Lactob_Lj965_NC_005355: hypothetical protein; PP_02316; phage(gi41179246)  conjugal transfer protein [*Lactobacillus plantarum* subsp. *plantarum* ST-III]. gi|308181191|ref|YP_003925319.1|; PP_02317  PHAGE_Lactob_Lj965_NC_005355: putative major head protein; PP_02318; phage(gi41179245)  PHAGE_Oenoco_phiS13_NC_023560: prophage protein; PP_02319; phage(gi589286250)  PHAGE_Lactob_Lj965_NC_005355: putative scaffold protein; PP_02320; phage(gi41179243)  PHAGE_Lactob_Lj965_NC_005355: putative minor head protein; PP_02321; phage(gi41179242)  hypothetical protein LPST_C2016 [*Lactobacillus plantarum* subsp. *plantarum* ST-III]. gi|308181198|ref|YP_003925326.1|; PP_02322  protein of hypothetical function DUF464 [*Lactobacillus plantarum* subsp. *plantarum* ST-III]. gi|308181199|ref|YP_003925327.1|; PP_02323  PHAGE_Lactob_Lj965_NC_005355: putative portal protein; PP_02324; phage(gi41179240)  PHAGE_Lactoc_PLgT_1_NC_031016: hypothetical protein; PP_02325; phage(gi100030)  PHAGE_Lactoc_PLgT_1_NC_031016: major tail protein; PP_02326; phage(gi100031)  PHAGE_Lactoc_PLgT_1_NC_031016: tail assembly protein; PP_02327; phage(gi100024)  hypothetical; PP_02328  hypothetical protein LC705_00865 [*Lactobacillus rhamnosus* Lc 705]. gi|258539056|ref|YP_003173555.1|; PP_02329  tRNA  tRNA  PHAGE_Lactob_LBR48_NC_027990: putative transcription regulator; PP_02330; phage(gi937456737)  PHAGE_Entero_phiFL2A_NC_013643: hypothetical protein; PP_02331; phage(gi281416518)  PHAGE_Lactob_Sha1_NC_019489: LP1-like protein; PP_02332; phage(gi418489797)  Prophage P2a protein 24 [*Lactobacillus plantarum* ZJ316]. gi|448821917|ref|YP_007415079.1|; PP_02333  PHAGE_Lactob_phig1e_NC_004305: hypothetical protein; PP_02334; phage(gi23455787)  PHAGE_Lactob_phig1e_NC_004305: hypothetical protein; PP_02335; phage(gi254854754)  PHAGE_Geobac_E2_NC_009552: hypothetical protein; PP_02336; phage(gi148747763)  PHAGE_Lactob_Sha1_NC_019489: phage protein; PP_02337; phage(gi418489843)  prophage P2a protein 15 [*Lactobacillus plantarum* 16]. gi|513841779|ref|YP_008121751.1|; PP_02338  hypothetical; PP_02339  PHAGE_Lactob_LBR48_NC_027990: putative transcription regulator; PP_02340; phage(gi937456713)  prophage Lp2 protein 12 [*Lactobacillus plantarum* subsp. *plantarum* ST-III]. gi|308181216|ref|YP_003925344.1|; PP_02341  hypothetical; PP_02342  PHAGE_Sulfit_pCB2047_A_NC_020858: XRE-like HTH transcriptional regulator; PP_02343; phage(gi472341910)  hypothetical; PP_02344  prophage P1 protein 10 [*Lactobacillus plantarum* WCFS1]. gi|380031634|ref|YP_004888625.1|; PP_02345  PHAGE_Lister_B054_NC_009813: gp41; PP_02346; phage(gi157325325)  PHAGE_Bacill_1_NC_009737: hypothetical protein; PP_02347; phage(gi155042958)  prophage P1 protein 6 [*Lactobacillus plantarum* WCFS1]. gi|380031630|ref|YP_004888621.1|; PP_02348  PHAGE_Lactob_KC5a_NC_007924: hypothetical protein; PP_02349; phage(gi90592577)  tRNA  PHAGE_Acidia_virus_NC_029316: hypothetical protein; PP_02350; phage(gi1002166880)  hypothetical; PP_02351  tRNA  attR CCGCCAAGAAGA  tRNA  tRNA  tRNA  PHAGE_Lactob_LBR48_NC_027990: putative integrase; PP_02352; phage(gi937456703) | 3e-39  8e-24  0.0  2e-06  3e-08  0.0  4e-28  0.0  4e-09  1e-06  1e-40  2e-103  2e-05  5e-06  4e-176  6e-29  0.0  2e-16  1e-39  7e-49  3e-32  4e-55  1e-33  5e-13  1e-26  4e-94  3e-32  2e-26  1e-91  6e-13  3e-26  1e-123  4e-136  2e-39  8e-19  0.0  2e-24  0.0  0.0  1e-42  7e-07  7e-09  1e-12  8e-70  1e-38  3e-70  6e-58  2e-13  0.0  2e-33  6e-53  0.0  2e-06  0.0  6e-55  8e-18  6e-10  2e-60  1e-28  0.0  2e-17  0.0  0.0  0.0  0.0  0.0  0.0  3e-53 |

*: Hits against Virus and prophage DB (marked in light blue) and hits against Bacterial DB or GenBank file (not marked)
