# Supplementary material for: In silico genomic insights into aspects of food safety and defense mechanisms of a potentially probiotic Lactobacillus pentosus MP-10 isolated from brines of naturally fermented Aloreña green table olives
Source: PLoS One. 2017 Jun 26;12(6):e0176801. doi: 10.1371/journal.pone.0176801 (PMC5484467; doi:10.1371/journal.pone.0176801)
Supplement: S3 Table — (DOC) [file pone.0176801.s004.doc]

| **Hit** | **Criteria** | **E-value** | **Percent Identity** | **ARO* accession** | **Model Cut-off in CARD database** |
| --- | --- | --- | --- | --- | --- |
| *Listeria monocytogenes mprF*  aminocoumarin resistant *alaS*  *lmrD*  *mfd*  *lmrB*  *lmrD*  *lmrB*  *lmrB*  *arlR*  *emeA*  Bifidobacteria intrinsic *ileS* conferring resistance to mupirocin  Bifidobacteria intrinsic *ileS* conferring resistance to mupirocin | Strict  Strict  Strict  Strict  Strict  Strict  Strict  Strict  Strict  Strict  Strict  Strict | 0  0  0  0  1.20891e-135  1.16187e-122  1.42201e-112  6.89032e-101  3.74685e-85  2.7358e-83  1.81776e-65  2.57762e-52 | 45  42  56  35  45  38  41  38  55  40  26  22 | ARO:3003770  ARO:003830  ARO:3002882  ARO:3003844  ARO:3002813  ARO:3002882  ARO:3002813  ARO:3002813  ARO:3000838  ARO:003551  ARO:3003730  ARO:3003730 | 1e-160  1e-50  1e-120  1e-100  1e-100  1e-120  1e-100  1e-100  1E-70  1e-80  1e-50  1e-50 |

**Table S3.** RGI results of AMR genes detected in *L. pentosus* MP-10 genome.

*: Antibiotic Resistance Ontology in CARD database.
