# Supplementary material for: In silico genomic insights into aspects of food safety and defense mechanisms of a potentially probiotic Lactobacillus pentosus MP-10 isolated from brines of naturally fermented Aloreña green table olives
Source: PLoS One. 2017 Jun 26;12(6):e0176801. doi: 10.1371/journal.pone.0176801 (PMC5484467; doi:10.1371/journal.pone.0176801)
Supplement: S4 Table — (DOC) [file pone.0176801.s005.doc]

**Table S4.** AMR detected in *L. pentosus* MP-10 genome by using hits with weak similarity “loose” in RGI software.

| **ARO*** | **Hit** |
| --- | --- |
| Gene modulating permeability to antibiotic  Rifampin resistance gene  Aminoglycoside resistance gene  Macrolide resistance gene  Lincosamide resistance gene  Pyrazinamide resistance gene  Isoniazid resistance gene  Chloramphenicol resistance gene  Gene modulating beta-lactam resistance  Streptogramin resistance gene  Antibiotic inactivation enzyme  Trimethoprim resistance gene  Antibiotic target replacement protein  Beta-lactam resistance gene  Tetracycline resistance gene  Antibiotic resistance gene cluster, cassette, or operon  Gene conferring antibiotic resistance via molecular bypass  Glycopeptide resistance gene  Fosfomycin resistance gene  Gene altering cell wall charge conferring antibiotic resistance  Polymyxin resistance gene  Elfamycin resistance gene  Antibiotic resistant gene variant or mutant  Gene involved in self resistance to antibiotic  Fluoroquinolone resistance gene  Antibiotic target protection protein  Aminocoumarin resistance gene  Gene modulating antibiotic efflux  Peptide antibiotic resistance gene  Antibiotic target modifying enzyme  Efflux pump conferring antibiotic resistance | 1  1  2  2  1  1  1  3  2  8  14  2  3  4  6  14  14  13  1  8  8  1  6  3  1  7  12  23  2  4  153 |

*: Antibiotic Resistance Ontology in CARD database.
